# Distinct coral environments shape the dynamic of planktonic *Vibrio* spp.

**DOI:** 10.1186/s40793-023-00532-7

**Published:** 2023-10-23

**Authors:** Wenbin Zhao, Xing Chen, Ronghua Liu, Peng Tian, Wentao Niu, Xiao-Hua Zhang, Jiwen Liu, Xiaolei Wang

**Affiliations:** 1 Frontiers Science Center for Deep Ocean Multispheres and Earth System, College of Marine Life Sciences, Ocean University of China, Qingdao, 266003 China; 2 Institute of Evolution and Marine Biodiversity, Ocean University of China, Qingdao, 266100 China; 3 Laboratory of Marine Biodiversity Research, Third Institute of Oceanography, Ministry of Natural Resources, 178 Daxue Road, Xiamen, 361005 China; 4 Nansha Islands Coral Reef Ecosystem National Observation and Research Station, Guangzhou, 510000 China

**Keywords:** *Vibrio* spp., Distribution patterns, Coral environments, Assembly mechanisms

## Abstract

**Background:**

Coral reefs are one of the most biodiverse and productive ecosystems, providing habitat for a vast of species. Reef-building scleractinian corals with a symbiotic microbiome, including bacteria, archaea, viruses and eukaryotic microbes, are referred to coral holobionts. Among them, coral diseases, mainly caused by *Vibrio* spp., have significantly contributed to the loss of coral cover and diversity. Habitat filtering across the globe has led to a variety structure of marine bacterial communities. Coral species, quantity and characteristics are significant differences between the Xisha Islands and Daya Bay (Guangdong Province). Thus, the *Vibrio* communities may be distinct between coral rich and poor areas.

**Results:**

Through comparison of *Vibrio* dynamics between coral-rich (Xisha Islands) and coral-poor (Daya Bay) locations, we uncovered differences in *Vibrio* abundance, diversity, community composition and assembly mechanisms associated with corals. The higher abundance of *Vibrio* in coral rich areas may indicate a strong interaction between vibrios and corals. *V. campbellii*, *Paraphotobacterium marinum* and *V. caribbeanicus* were widely distributed in both coral rich and poor areas, likely indicating weak species specificity in the coral-stimulated growth of *Vibrio*. Random-forest prediction revealed *Vibrio* species and *Photobacterium* species as potential microbial indicators in the coral rich and coral poor areas, respectively. Ecological drift rather than selection governed the *Vibrio* community assembly in the Xisha Islands. Comparatively, homogenizing selection was more important for the Daya Bay community, which may reflect a role of habitat filtration.

**Conclusion:**

This study revealed the different distribution pattern and assembly mechanism of *Vibrio* spp. between coral rich and poor areas, providing the background data for the research of *Vibrio* community in coral reef areas and may help the protection of coral reef at the biological level. The main reasons for the difference were different number and species of corals, environmental (e.g., temperature) and spatial factors. It reflected the strong interaction between *Vibrio* and corals, and provided a new perspective for the investigation of *Vibrio* in coral reef ecosystem.

**Supplementary Information:**

The online version contains supplementary material available at 10.1186/s40793-023-00532-7.

## Background

Coral reef is the most biologically diverse and productive ecosystem on earth, providing habitat for a vast array of species that build up a three-dimensional reef matrix by the calcium carbonate skeletons secreted from corals over time [1]. Though covering a small area of the ocean floor, coral reefs sustain a third of all described marine species [2]. For instance, the biomass of resident reef fish averages ~ 1000 kg/hectare [3]. Corals are usually symbiotic with algae, such as *zooxanthellae* for nutrient cycle to maintain the stability and growth of the reef [4]. Prokaryotic microorganisms also play important roles in the coral reef ecosystem, such as microbial-coral diseases, and interactions between coral symbiotic algae and microorganisms [5–7]. Bacteria can contribute to the physiology and health of the coral host through nutrient acquisition and metabolic cycling [8]. Shnit-Orland and Kushmaro have found that 25–70% of coral mucus-associated bacteria exhibited antibacterial activity, suggesting that coral mucus-associated bacteria may protect their coral hosts against pathogens [9]. Furthermore, nitrogen-cycling microbes may be critical to the stability of coral-algal symbiosis and the function of holobiont, and disturbances of microbial nitrogen cycling may be closely associated with coral bleaching and disease [10]. In addition, microbial processes can influence the resilience of coral reef ecosystems by influencing the colonization of larvae through chemical cues [11]. Thus, the diversity and metabolic activity of surrounding microorganisms in seawater may provide important solutions to potentially protect coral reefs.

Corals and their symbiotic algae may also exert influences on microbes in the surrounding seawater. For example, corals release mucus affecting microbial abundance and composition [12]. Coral mucus is rich in organic matter and nutrients, with concentrations of total organic carbon and nitrogen being 2–4 times higher than that of the surrounding seawater [13, 14]. Previous studies have shown a significant increase in microbial abundance in seawater with coral mucus presence [14]. Moreover, the exudates of coral symbiotic algae can transport sugars into the environment, promoting growth of planktonic bacteria [15], particularly *Vibrio* including pathogenic species such as *Vibrio cholerae* [16]. Comparison of microorganisms from four different environmental niches (*Acropora palifera*, *Acropora aspera*, sandy substrate and open water) in the Great Barrier Reef has shown increased occurrence frequency of *Vibrio*, *Pseudoalteromonas* and *Alteromonas* in coral seawater niches [17]. In a shallow reef in St. John, U.S. Virgin Islands, bacteria and archaea in the surrounding seawater showed significant temporal and spatial variations, but only few vibrios were present in the total community compositions [18]. However, the ecological distribution and community dynamics of *Vibrio* spp. in coral surrounding seawaters are still unclear.

*Vibrio* spp. is a group of Gram-negative rod-shaped bacteria belonging to the class *Gammaproteobacteria* with facultative fermentative metabolisms [19], which is highly heterogeneous and abundant in various aquatic environments [20, 21]. At present, the research on *Vibrio* in coral reefs mainly focuses on coral diseases, such as *V. coralliilyticus* causing white syndrome, *V. alginolyticus* causing yellow band disease and *V. natriegens* causing white spot disease [7]. *Vibrio* multiplies rapidly, has a short generation time, and responds quickly to nutrient pulses likely due to their highly plastic genomes and wide metabolic ranges [21]. Meanwhile, vibrios can be subject to predation by bacteriophages and protozoa, such as *Vibrio alginolyticus* and *Vibrio natriegens* [22, 23]. Based on these knowledges, the biogeochemical roles of *Vibrio* were underestimated for a long time. *Vibrio* community may rapidly respond to nutrient pulses produced by corals, which may cause the community dynamics in coral reef ecosystems differed from other marine environments. Previous study has found that *V. hyugaensis*, *V. owensii* and *V. harveyi* were the dominant species, and the influence of temperature on these species was evaluated in the coral reefs of Ishigaki (Japan) [24]. As a typical coral conservation area, the *Vibrio* community of Dongshan Bay (Fujian, China) was dominated by *V. fortis*, *V. natriegens* and *V. pomeroyi*, revealing a distance-decay pattern spanning four seasons [19]. The distribution pattern of corals in different coral reef environments may dominate the dynamics and assembly mechanisms of *Vibrio* community. However, the correlations between *Vibrio* spp. and corals needs to be further studied.

Corals can be divided into "reef" and "non-reef" coral communities, and the latter is characterized by the inability to accumulate calcium carbonate [25]. The coral species and the local reef environment can influence the composition of microbial community [26]. The majority of China’s coral reefs are found in the South China Sea, whereas only limited and scattered subtidal coral communities are found from the west coast of Leizhou Peninsula (Guangdong, China) to Dongshan Bay (Fujian, China) [27, 28]. The Xisha Islands (coral rich areas) mainly consisting of 36 atolls, is one of the four groups of islands located in the South China Sea, with plentiful coral reefs [29–31]. A total of 213 species of scleractinian corals (belonging to 43 genera and 16 families) have been discovered therein [32]. Daya Bay (coral poor areas) is one of the few coastal bays in China with coral distribution [33]. The corals scattered in the Daya Bay mainly appear in the form of communities and have not developed into coral reefs due to climate constraints [34]. A total of 44 species (belonging to 17 genera and 9 families) and 5 unclassified species of scleractinian corals are found there [35]. At present, the studies on microbial dynamics in Daya Bay and Xisha Islands mainly focus on the dynamic of total bacteria or the changes of culturable bacteria [36, 37], with no attention on the dynamic changes of the vibrionic community. Given that different environments affect microbial colonization [26], *Vibrio* may have different dynamic characteristics in these two coral environments. In this study, the dynamics of *Vibrio* communities in seawater from the coral rich and poor areas were investigated by qPCR and high-throughput sequencing techniques. The key factors affecting the community structure and assembly mechanism of *Vibrio* community in distinct coral environments were illustrated.

## Methods

### Water sampling and physicochemical parameter determination

**Fig. 1:**
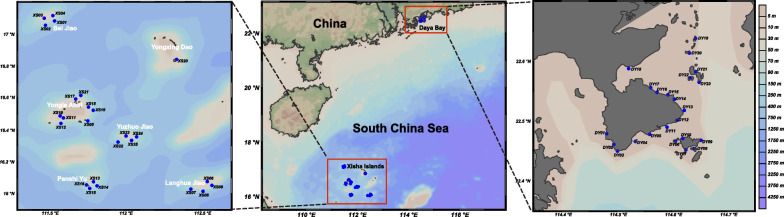
Map showing locations of study area and sampling sites. [The map was created using Ocean Data View (version 5.5.2; R. Schlitzer, Ocean Data View, https://odv.awi.de, 2021.)]

To explore the planktonic *Vibrio* communities in distinct coral areas, waters from the Xisha Islands and Daya Bay were collected by the *R/V Yuezhanyuke10* from a total of 48 sites during 31 August and 30 September, 2020 (Fig. 1). A total of 85 samples were collected, and detailed information of the samples was recorded in Additional file 1: Table S1. At each sample, 1 L (L) of water was filtered through polycarbonate membranes (Millipore Corporation, Billerica, MA, USA) 3-μm for particle-associated (PA) and 0.22-μm for free-living (FL) microbes, respectively. All filters were stored in liquid nitrogen onboard and transferred to − 80 °C in the laboratory until DNA extraction. The physicochemical parameters of waters were determined in situ and in the laboratory. CTD was used to monitor the water chemistries such as temperature, salinity, pH, dissolved oxygen (DO) and suspended solid (SS). Water samples for dissolved inorganic nutrients (NO_2_^–^, NO_3_^–^ and NH_4_^+^), and Chlorophyll a (Chl* a*) analyses were collected according to our previous work [38]. Dissolved inorganic nitrogen (DIN) were calculated as the sum of NO_3_^−^, NO_2_^−^ and NH_4_^+^ [39]. After acid fumigation to remove the carbonate fraction, total nitrogen (TN) was determined by an elemental analyzer (vario MICRO cube EA, Elementar, Germany) which interfaced with a continuous flow isotope ratio mass spectrometer (Isoprime IRMS, Elementar, Germany) [40, 41]. Detection of the total phosphorus (TP) was conducted followed the description of Church et al. [42] and Varol et al. [43]. Additionally, the identification and abundance of coral species were provided by the Laboratory of Marine Biodiversity Research, Third Institute of Oceanography, Ministry of Natural Resources. Briefly, three transects (50 m in length) of different depths (5, 10 and 15 m) were set up at each sampling sites. A SCUBA diver performed Point Intercept Transect (PIT) video sampling at each transects, and another diver took close-up photographs of various corals under the tapeline and collected some specimens for species identification according to standard procedures [32]. Video transects were analyzed in the laboratory using a point sampling technique according to the taxonomic criteria [32], and the coral close-up photographs and the coral specimens were used to assist the species identification. The number of coral species were calculated based on the identification results.

### DNA extraction and quantitative PCR (qPCR)

Genomic DNA was extracted according to the previous report [38]. The extracted DNA was resuspend using 50 μL TE buffer (1 M Tris–HCl, 0.5 M EDTA, pH 8.0) and preserved at – 80 ℃ until use. Through 16S rRNA gene-targeted quantitative PCR (qPCR) with SYBR-green detection, the absolute abundance of total *Vibrio* on 3- and 0.22-μm-pore-size membranes were tracked. Briefly, the StepOnePlus real-time PCR system (Applied Biosystems) and StepOne software version 2.2 was operated to performed the qPCR. V567F (5′-GGCGTAAAGCGCATGCAGGT-3′) and V680R (5′-GAAATTCTACCCCCCTCTACAG-3′) primers were utilized to quantify the *Vibrio* spp. [20, 44]. The reaction mixture and cycling conditions referred to the descriptions of Wang et al. [38] and Liang et al. [45]. The standard was prepared using 16S rRNA gene of *Vibrio rotiferianus* WXL191 (our laboratory) and the preparation methods followed our previous work [38]. qPCR was carried out in triplicates and all amplification efficiencies were between 95 and 105% with R^2^ values > 0.99.

### High-throughput sequencing and reads processing

The *Vibrio*-specific 16S rRNA gene primers V169F (5′-GGATAACC/TATTGGAAACGATG-3′) and V680R (5′-GAAATTCTACCCCCCTCTACAG-3′) were utilized to determine the composition of *Vibrio* community [45]. The reaction mixture and cycling conditions followed the descriptions of Wang et al. [38]. After confirming positive amplification, sequencing was performed on the Illumina Miseq PE300 platform at Majorbio Bio-Pharm Technology Co., Ltd. (Shanghai, China). Raw reads were trimmed with FASTP, removing those short length sequences (< 100 bp) and low quality (< 20) [46]. Paired-end DNA sequences were then joined using FLASH with at least a 10-bp overlap and < 5% mismatches [47]. Moreover, chimeric sequences, barcode sequences and primers were removed using the DADA2 plug-in of software QIIME2 [48, 49]. Then, the sequences of *Vibrio* spp. were clustered into operational taxonomic units (OTUs) by QIIME2 software. The taxonomy of each OTUs representative sequences were assigned by local-blast against the SILVA v138 database (a minimum support threshold of 70%), and they were reassigned against the EzBioCloud database (https://www.ezbiocloud.net/) to acquire a more accurate taxonomic identification.

### Statistical analysis

To minimize biases associated with sequencing coverage, the number of sequences for each sample was homogenized to the lowest number (32,029 reads) by running a script in R software. The differences of between separate groups of physicochemical parameters were calculated by Dunn's test. Alpha (α)-diversity was indicated by Shannon, Chao 1 and Simpsoneven indices, which were calculated using the “vegan” package. The differences between separate groups of samples were examined by *t*-test. For Beta (β)-diversity, the non-metric multidimensional scaling (NMDS) was performed at the OTU level by Canoco 5.0 software [50]. The subsequent analysis of similarities (ANOSIM) was performed using the anosim function with 999 permutations in “vegan”. The niche widths of the most thirty abundant OTUs were calculated by “vegan” and “spaa”. The random forest machine learning was performed with “randomForest” package in R. We used variation partition analysis (VPA), performed by the “vegan” package, to estimate the relative contributions of geographic distance, environmental factors and coral species to *Vibrio* community structure. To reveal the relationship between environmental factors and microbial communities, Mantel test based on Pearson’s correlations was carried out by the “ggcor” package. To determine the relationship between *Vibrio* species, α-diversity, the abundance of *Vibrio* and various factors, the software IBM SPSS Statistica (v 23.0.) was operated to calculate Spearman's rank correlation coefficient. Additionally, a null model analysis was carried out to quantify the relative contributions of different ecological processes, which was calculated using the “picante” package.

## Results

### Environmental conditions and coral distribution

Water temperature, salinity, SS, Chl *a*, TN, TP and pH varied significantly between Daya Bay and Xisha Islands (*P* < 0.05; Additional file 1: Fig. S1). Compared to the Daya Bay, the pH (8.11 ± 0.06), salinity (33.85 ± 0.07; practical salinity units [PSU]), TP (0.009 ± 0.003 mg L^−1^), SS (2.61 ± 0.60 mg L^−1^) and Chl *a* (0.38 ± 0.34 μg L^−1^) were significantly lower in the Xisha Islands. However, the Xisha Islands featured higher temperature (30.84 ± 0.41 ℃) and TN concentration (0.23 ± 0.07 mg L^−1^; Table 1). The abundance and number of corals species was higher in Xisha Islands than in Daya Bay. All corals were assigned to 15 families and 1 unclassified family, with 9 families of corals in Daya Bay, and 15 families and the unclassified coral in Xisha Islands. The number of corals in the Xisha Islands was nearly 6 times than that in Daya Bay. Difference in dominant corals was observed between Daya Bay and Xisha Islands (Additional file 1: Fig. S2). *Poritidae* was the most dominant coral in the Daya Bay, followed by *Merulinidae* and *Acroporidae*, whereas the dominant corals of the Xisha Islands were *Merulinidae*, followed by *Poritidae*, *Acroporidae* and *Pocilloporidae*. Several kinds of corals (i.e., *Pocilloporidae*, *Oulastreidae*, *Coscinaraeidae*, *Fungiidae*, *Astrocoeniidae*, *Lobophylliidae* and the unclassified one) were unique at the Xisha Islands. Detailed information on environmental parameters and coral numbers are provided in Additional file 1: Tables S2 and S3, respectively.

**Table 1:** Environmental parameters of the sampling sites

Characteristic	Daya Bay			Xisha Islands		
	Mean ± SD	Min	Max	Mean ± SD	Min	Max
Longitude (°E)	114.59 ± 0.05	114.48	114.65	111.87 ± 0.34	111.47	112.57
Latitude (°N)	22.52 ± 0.06	22.45	22.64	16.48 ± 0.37	16.01	17.12
Temperature (℃)	27.11 ± 2.80	21.80	31.40	30.84 ± 0.41	30.30	31.70
Salinity (PSU)	34.00 ± 0.27	32.98	34.37	33.85 ± 0.07	33.70	34.05
pH	8.13 ± 0.04	8.05	8.19	8.11 ± 0.06	8.02	8.26
DO (mg L^−1^)	6.39 ± 0.47	5.01	7.53	6.52 ± 0.91	5.28	8.23
Chl *a* (μg L^−1^)	2.75 ± 2.33	0.13	11.55	0.38 ± 0.34	0.09	1.57
NH_4_^+^ (μmol L^−1^)	0.02 ± 0.01	0.01	0.06	0.02 ± 0.01	0.01	0.06
DIN (μmol L^−1^)	0.02 ± 0.01	0.01	0.07	0.02 ± 0.01	0.01	0.08
TN (mg L^−1^)	0.13 ± 0.06	0.05	0.34	0.23 ± 0.07	0.09	0.41
TP (mg L^−1^)	0.014 ± 0.003	0.008	0.027	0.009 ± 0.003	0.006	0.018
SS (mg L^−1^)	12.28 ± 3.10	7.10	20.00	2.61 ± 0.60	1.20	3.60

*DO* dissolved oxygen, *Chl a* chlorophyll a, *DIN* dissolved inorganic nitrogen, *TN* total nitrogen, *TP* total phosphorus, *SS* suspended solid

### Total *Vibrio* abundance

**Fig. 2:**
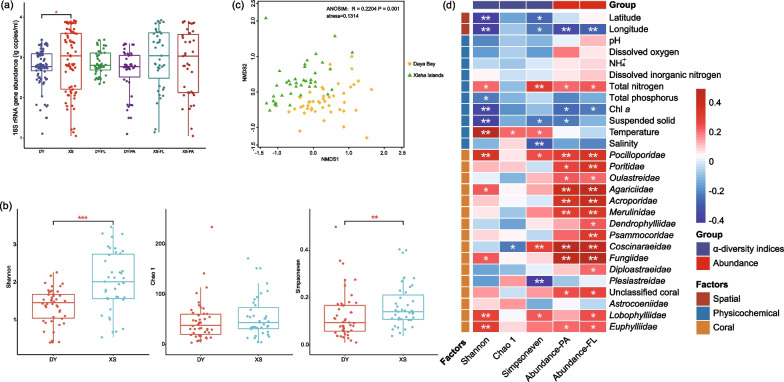
The abundance and diversity of *Vibrio* spp. **a** Total *Vibrio* abundance (lg copies/ml) across all samples and their differences among different groups. *DY* Daya Bay, *XS* Xisha Islands, *PA* particle-associated group, *FL* free-living group. *P < 0.05. **b** α-Diversity indices of *Vibrio* community. Student’s t-test showed significant difference between DY and XS. **P < 0.01; ***P < 0.001. **c** Nonmetric multidimensional scaling ordination of vibrios based on the Bray–Curtis dissimilarity. **d** Spearman correlations between *Vibrio* spp. abundance, α-diversity indices and the physicochemical parameters. *P < 0.05; **P < 0.01

*Vibrio* abundance in the Xisha Islands (2.92 ± 0.78 lg copies/mL) was significantly higher than that in Daya Bay (2.76 ± 0.46 lg copies/mL; *P* < 0.05; *t*-test), representing an obvious spatial distribution pattern (Fig. 2a). Meanwhile, both the average abundance of FL and PA *Vibrio* were higher in Xisha Islands than in Daya Bay, although the difference was not significant (Fig. 2a). Detailed information of *Vibrio* abundance was listed in Additional file 1: Table S4. This variation of *Vibrio* abundance between the two areas largely related to the corals. In both the two areas, the abundance of FL and PA *Vibrio* showed significantly positive correlations to several kinds of corals (i.e., *Pocilloporidae*, *Poritidae*, *Oulastreidae*, *Agariciidae*, *Acroporidae*, *Merulinidae*, *Coscinaraeidae*, *Fungiidae*, *Euphylliidae* and unclassified corals; *P* < 0.05 or 0.01; Fig. 2d). The *Vibrio* abundance was positively correlated with *Pocilloporidae*, *Poritidae*, *Agariciidae*, *Acroporidae*, *Merulinidae*, *Coscinaraeidae* and *Fungiidae* in Xisha Islands, whereas no correlation was found in Daya Bay (*P* < 0.05 or 0.01; Additional file 1: Fig. S3).

### Diversity estimators of *Vibrio* spp.

To analyze the diversity of the *Vibrio* community, 16S rRNA gene amplicons were sequenced by the Illumina Miseq PE300 platform. The high-throughput sequencing yielded 4,412,547 clean reads from 85 samples ranging from 32,029 to 82,544 reads per sample. After discarding the singletons and rarefaction, 32,029 sequences per sample were left. The total sequences were clustered into 549 operational taxonomic units (OTUs) based on a 97% sequence similarity level. And, the coverages of all samples were close to 99.9%, indicating that the current sequences could represent the real situation of *Vibrio* community in the studies sites.

For α-diversity indices, evenness, richness and diversity of *Vibrio* were variable between the two areas (Fig. 2b). Although the richness (Chao 1) had no significant difference between Daya Bay and Xisha Islands, *Vibrio* evenness in the Xisha Islands was higher than that in Daya Bay (Simpsoneven, *t*-test, *P* < 0.01), leading to a higher diversity level (Shannon, *t*-test, *P* < 0.001). Overall, correlation analysis demonstrated that *Pocilloporidae*, *Agariciidae*, *Coscinaraeidae*, *Fungiidae*, *Plesiastreidae*, *Lobophylliidae* and *Euphylliidae* were related to changes in α-diversity (Fig. 2d). However, the variation of α-diversity in the Xisha Islands showed association with *Acroporidae*, *Psammocoridae*, *Coscinaraeidae* and *Astrocoeniidae*, and the changes in the Daya Bay were associated with *Poritidae*, *Acroporidae*, *Psammocoridae* and *Plesiastreidae* (Additional file 1: Fig. S3). We also analysed the β-diversity among all samples. NMDS analysis based on Bray–Curtis distances showed spatial separation of *Vibrio* compositions between the two regions (Analysis of similarity [ANOSIM]; R = 0.2204; *P* = 0.001; Fig. 2c). The β-diversity of *Vibrio* community in the Xisha Islands was higher than that in Daya Bay (*P* < 0.001; Additional file 1: Fig. S4). Detailed information on the diversity parameters was recorded in Additional file 1: Table S4.

### The community compositions of *Vibrio* spp.

**Fig. 3:**
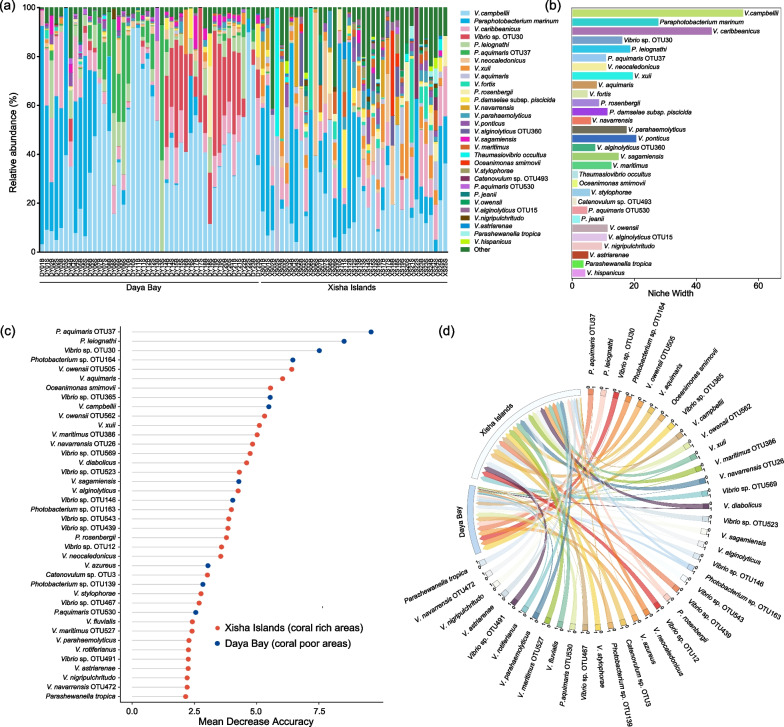
Characteristics of microbial communities. **a** Compositions of *Vibrio* spp. at the species level. **b** The niche widths of the top 30 OTUs. **c** The most important 40 OTUs reducing the uncertainty in the prediction of geography (Daya Bay and Xisha Islands) based on their mean decrease in accuracy. **d** Relative proportions of the most important 40 OTUs in the Xisha Islands and Daya Bay

After re-annotated against the EzBioCloud database, *Vibrionaceae* accounted for 52.60% of the total sequences, and *Vibrio* spp. accounted for 78.92% of *Vibrionaceae* sequences. The abundance of the top 30 OTUs accounted for 92.85% of all species (Fig. 3a). Among them, OTU30 and OTU493 were annotated to the genera *Vibrio* and *Catenovulum*, respectively.

*V. campbellii* occupied the highest relative abundance across all samples (31.03%), and was more abundant in the Daya Bay than in the Xisha Islands (Additional file 1: Fig. S5;* P* < 0.001). Furthermore, *Vibrio* sp. OTU30, *P. leiognathi*, *P. aquimaris* OTU37 and *V. sagamiensis* also had higher abundance at the Daya Bay (*P* < 0.05), whereas *V. neocaledonicus*, *V. xuii*, *V. aquimaris*, *V. fortis*, *V. navarrensis*, *V. parahaemolyticus*, *V. ponticus*, *V. alginolyticus*, *V. maritimus*, and *V. stylophorae* were more abundant at the Xisha Islands (*P* < 0.05; Additional file 1: Fig. S5). Additionally, the Daya Bay could be divided into inside (DY01 to DY13) and outside sites (DY14 to DY23) according to the geographical location (Fig. 1). Five species had significant differences between the inside and outside sites by Dunn's test, i.e., *Paraphotobacterium marinum* (*P* < 0.01), *Vibrio* sp. OTU30 (*P* < 0.001), *P. aquimaris* OTU37 (*P* < 0.01), *V. ponticus* (*P* < 0.05) and *P. aquimaris* OTU530 (*P* < 0.01). Further, we calculated the niche widths of the top 30 OTUs to explore their adaptation. *V. campbellii* has the largest niche width, which may have the most adaptable to this environment (Fig. 3b).

To distinguish these two different coral environments, a random-forest machine-learning model was established to find the important features that could distinguish the coral rich and poor areas (Fig. 3c). The model had the highest accuracy (lowest out of bag (OOB) estimated error rate) when the number of top important features reached 40 OTUs (Additional file 1: Fig. S6). Therefore, the most characteristic OTUs (top 40) were selected as a group of biomarkers to distinguish the two habitats, such as *P. aquimaris* OTU37 representing coral poor area and *V. owensii* OTU505 representing coral rich area (Fig. 3c). Meanwhile, the relative abundance of 40 characteristic OTUs showed differences in the two regions (Fig. 3d).

### Various factors governing the spatial dynamics of *Vibrio* spp.

**Fig. 4:**
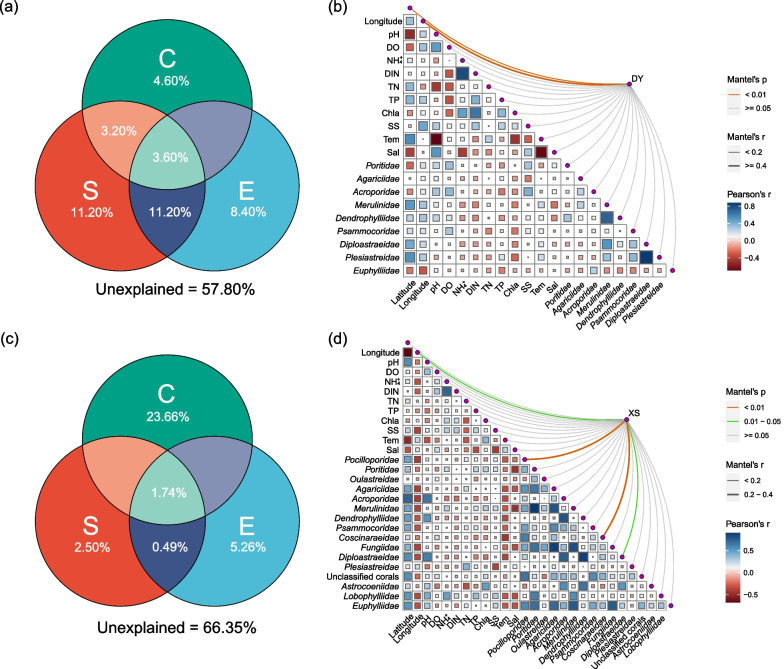
Effects of various factors on *Vibrio* community. **a**, **c** Variation partitioning analyses of the *Vibrio* community composition between biotic and abiotic variables. **a**, Daya Bay; **c**, Xisha Islands. *E* environmental factors, *S* spatial factors, *C* coralline factors. **b**, **d** Relationships between microbial community and other factors in different areas using Mantel test. **b** Daya Bay (DY); **d** Xisha Islands (XS). *DO* dissolved oxygen, *DIN* dissolved inorganic nitrogen, *TN* total nitrogen, *TP* total phosphorus, *Chl a* chlorophyll a, *SS* suspended solid, *Tem* temperature, *Sal* salinity

To explore the key drivers shaping the *Vibrio* community, biotic and abiotic correlations were analyzed by VPA (Fig. 4). For Daya Bay, the pure effect of spatial factors (11.20%) was significantly greater than environmental (8.40%) and coralline (4.60%) variables (Fig. 4a), whereas the pure effect of coralline variables (23.66%) was greater than environmental (5.26%) and spatial (2.50%) factors in the Xisha Islands (Fig. 4c). However, 57.80% and 66.35% of the variation in Daya Bay and Xisha Islands were unexplained, respectively. Mantel test was used to evaluate the specific factors affecting *Vibrio* community structure. The *Vibrio* community in the Xisha Islands was significantly correlated with *Pocilloporidae*, *Coscinaraeidae* and *Diploastraeidae* (*P* < 0.05 or *P* < 0.01; Fig. 4). Detailed parameters were provided in Additional file 1: Table S5.

**Fig. 5:**
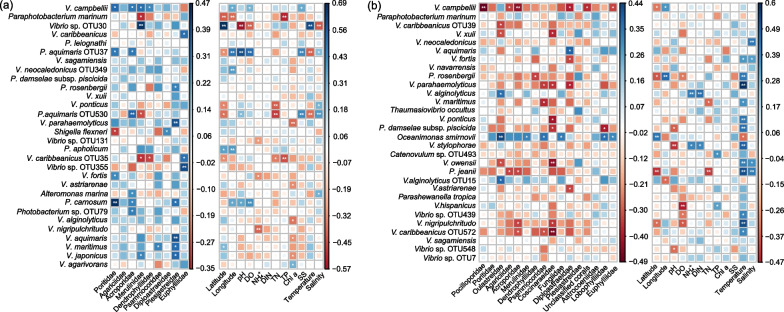
The correlations of the most abundant 30 OTUs with abiotic and biotic factors. **a** Daya Bay; **b** Xisha Islands. *P < 0.05; **P < 0.01

The Spearman’s rank correlation coefficients between the top 30 OTUs and factors were calculated (Fig. 5). As the most abundant specie in these two areas, *V. campbellii* showed significantly positive correlations with *Poritidae*, *Acroporidae*, *Merulinidae*, *Dendrophylliidae*, DO and SS in Daya Bay (*P* < 0.05; Fig. 5a), whereas negatively correlated to *Pocilloporidae*, *Agariciidae*, *Acroporidae*, *Diploastraeidae*, *Euphylliidae* and the unclassified corals in Xisha Islands (*P* < 0.05 or 0.01; Fig. 5b). The abundant species in the Daya Bay, i.e., *Vibrio* sp. OTU30, was positively correlated with *Merulinidae* and temperature, and significantly negative to pH, DO and salinity (*P* < 0.01; Fig. 5a). Similarly, *V. neocaledonicus* in the Xisha Islands only had positive correlation with salinity (*P* < 0.01; Fig. 5b).

### Assembly process of the *Vibrio* community

**Fig. 6:**
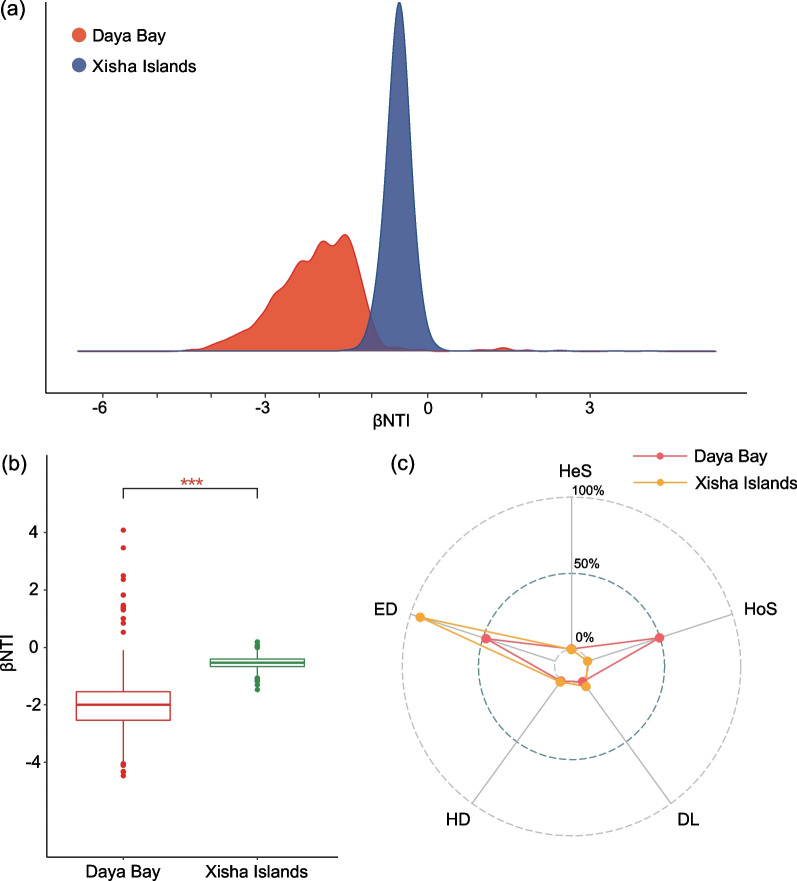
Community assembly mechanisms of *Vibrio* communities. **a** The distribution of βNTI in different groups. **b** Comparison of βNTI between Daya Bay and Xisha Islands. ***P < 0.001. **c** The assembly processes of *Vibrio* communities with the null model. *HeS* Heterogeneous selection, *HoS* Homogenizing selection, *DL* Dispersal limitation, *HD* Homogenizing dispersal, *ED* Ecological drift

We used the βNTI metric to quantify the relative importance of deterministic (|βNTI|> 2) or stochastic (|βNTI|< 2) factors to community structure. According to the description of Stegen et al., ecological processes are divided into deterministic processes (i.e., heterogeneous and homogeneous selection) and stochastic processes (i.e., homogenizing dispersal, ecological drift and dispersal limitation) [51]. There was a significant variation in βNTI values between Daya Bay and Xisha Islands via *t*-test (*P* < 0.001; Fig. 6b). The βNTI values of *Vibrio* in the Daya Bay were between − 4 and − 1, indicating that both deterministic and stochastic processes had an impact on *Vibrio* community structure. The βNTI values of *Vibrio* in the Xisha Islands were mostly between − 2 and 2, indicating the dominant effects from stochastic processes (Fig. 6a). The community of vibrios in the Daya Bay were jointly governed by ecological drift (47.9%) and homogeneizing selection (49.7%). It was noteworthy that ecological drift contributed the largest fraction (93.4%) to the community structure in the Xisha Islands (Fig. 6c).

## Discussion

Coral reefs are one of the most productive and biologically diverse ecosystems on earth. The distribution patterns of microorganism in distinct coral environments often show differences [52, 53]. *Vibrio* is related to the health of corals, so that the dynamics of *Vibrio* spp. may be various between different coral environments. Previous reports on the ecological distribution of *Vibrio* spp. mostly focused on estuarine, bay, or general marine environments [19, 38, 45, 54, 55]. Here, we assessed the abundance, distribution and community assembly mechanisms of *Vibrio* between coral rich (Xisha Islands) and coral poor (Daya Bay) environments. This is the first investigation regarding *Vibrio* distribution patterns and assembly mechanism in relation to coral species and density. Our study provides a foundation for study of the distribution dynamics of *Vibrio* associated with coral, and provides data support for coral conservation and restoration.

### High *Vibrio* abundance in the coral rich areas may indicate their strong interaction with corals

Microbes in coral surrounding seawater are significantly different from those in ordinary seawater or sediments [17]. In our study, the average *Vibrio* abundance in coral reef areas was 1.57 × 10^3^ copies/mL (Additional file 1: Table S4), higher than the value (1.17 × 10^3^ copies/mL) in the South China Sea [55]. These abundance heterogeneities may be attributed to the presence of corals, in which high coral productivity [5] may provide nutrients for the growth of *Vibrio*. Moreover, the abundance of *Vibrio* in the Xisha Islands was higher than that in the Daya Bay (Fig. 2a, b). Differences in the number of corals may also affect *Vibrio* abundance. The number of corals in the Xisha Islands was nearly six times higher than that in the Daya Bay (Additional file 1: Fig. S2). Many interaction processes between microbes and corals have been reported. Corals can release dissolved and particulate organic matter into surrounding seawater in the form of coral mucus, which could aid heterotrophic feeding [13, 56], resulting higher bacterial abundance in coral mucus than in the surrounding seawater [57, 58]. *Vibrio* populations are able to rapidly respond to nutrient enrichment and use various quorum sensing (QS) signal molecules to regulate the process to colonize coral under different environmental conditions [59]. We also found positive correlation between the abundance of *Vibrio* and several corals in the Xisha Islands, while no correlation was found in the Daya Bay (Additional file 1: Fig. S3). Pollock et al. provided evidence of coral-microbe phylosymbiosis, in which the phylogeny of coral was related to the composition and richness of the coral microbiome [60]. Therefore, a higher *Vibrio* abundance may be required to maintain interactions with abundant corals in the Xisha Islands. In addition, coral bleaching occurred in the Xisha Islands in 2020 may be another reason for the high *Vibrio* abundance [32]. Bleached corals release twice as much particulate organic carbon (POC) and particulate nitrogen (PN) than normal corals [61], and coral pathogenic microbes may respond rapidly to these nutrient pulses [62, 63]. It has been reported that vibrios usually can respond to fluctuations in nutrient concentrations and show a tendency of explosive reproduction [21]. Indeed, during the 2015 to 2016 global coral bleaching event, the abundance of potential coral pathogens (such as *Vibrio* spp.) in the southern South China Sea increased from about 0.28 to ~ 52.92% [64].

Temperature and TN are also found to affect *Vibrio* abundance. The importance of temperature in regulating *Vibrio* abundance has been widely reported [44, 45]. For TN, a higher concentration was found in Xisha Islands than in Daya Bay (Additional file 1: Fig. S1). In general, the nitrogen cycle is of great importance to coral environments. Coral symbiotic algae can provide photosynthates to the coral host, but these photosynthates have been referred to as "junk food" due to a high C:N ratio and additional nitrogen is required to sustain coral growth [65]. The high efficiency of nitrogen absorption by coral holobiont enables this symbiotic relationship to effectively utilize nitrogen compounds in the surrounding seawater [10]. In addition, strains of *Vibrio* spp. and *Photobacterium* spp. showed the ability of nitrogen fixation [66–68]. A study have found that *Vibrio* spp. and *Photobacterium* spp. may be involved in the nitrogen-fixing process in the sediments of the South China Sea, and *Vibrio* spp. may also be involved in the dissimilatory nitrate reduction to ammonium [69]. Thus, *Vibrio* may co-exist with coral by participating in the nitrogen cycle process. We also found that the abundance of PA *Vibrio* in the Xisha Islands was higher than that in Daya Bay (Fig. 2a), which may be related to the particulate matter or attachment environment provided by corals [56].

### Corals may weaken the species specificity of *Vibrio* spp. in the Chinese coral reef areas

Microbes may have strong adaptabilities facilitating their wide spread across various marine environments [70]. In the non-coral areas, including the South China Sea, the Chinese northern marginal seas and the Changjiang estuary, 160, 74 and 40 *Vibrio* OTUs were reported, respectively [38, 45, 55]. Comparatively, in this study, we obtained a total of 549 OTUs, suggesting that the presence of coral significantly increase the diversity of *Vibrio* spp. due to weak species-specific stimulation. Indeed, the abundant group in the coral-poor area, including *V. campbellii*, *Paraphotobacterium marinum* and *V. caribbeanicus*, were also dominant in the coral-rich area, corresponding their high niche widths. Predominance of these species in both areas may indicate their strong environmental adaptablities. *V. campbellii* is widely distributed, even in the oligotrophic deep sea, and can acquire gene horizontally to enhance adaptation of environment [70]. As a common pathogen of shrimp [71], *V. campbellii* encodes a sugar-specific porin in the outer membrane responsible for the transport of chitooligosaccharides, allowing it to grow rapidly in aquatic environments with chitin as a nutrient [72]. The environmental adaptability of *V. campbellii* was also reflected by its strong ability to degrade macromolecule substances [70], possession of quorum sensing for interbacterial cell killing and biofilms formation [73], and expression of *rpoS* ortholog gene (*rpoS1*) and *rpoS* xenolog gene (*rpoS2*) [74]. *Paraphotobacterium marinum* possesses the activity of a variety of enzymes including alkaline phosphatase, esterase (C4), leucine arylamidase and acid phosphatase, which can utilize D-Glucose and D-maltose, and reduce nitrate to nitrite [75]. Similarly, *V. caribbeanicus* also has the activity of various enzymes such as alkaline phosphatase and esterase lipase (C8), and can use a variety of amino acids and sugars [76]. The multiple enzyme activities and strengthening substrates utilization capabilities of *Paraphotobacterium marinum* and *V. caribbeanicus* may favor their growth in two areas.

It has been reported that the community compositions of *Vibrio* usually separated in various marine environments [21, 77]. In this study, except *V. campbellii*, *Paraphotobacterium marinum* and *V. caribbeanicus*, the rest species (e.g., rare taxa) showed differences in their relative abundances between coral rich and poor areas (Fig. 3), which may be related to habitat differentiation. We obtained 40 indicator species as marker species that can distinguish the two coral environments using random-forest machine-learning model (Fig. 3c). Among the indicator species in the Daya Bay, temperature may affect *P. aquimaris* OTU37 and OTU530. The growth temperature of *P. aquimaris* is 10–25 ℃, and cannot grow at 30 and 37 ℃ [78], whereas the temperature of the Xisha Islands is more than 30 ℃. Additionally, *P. leiognathi* was isolated from the luminescent organs of fishes in family *Leiognathidae* [79]. Fishes of family *Leiognathidae* are common fish resources in the southern coast of China, and studies have found that eggs of family *Leiognathidae* account for the largest proportion of all fish eggs in the Daya Bay [80, 81]. Thus, the special fish resources and temperature may explain why most of the indicator species in the Daya Bay are of the *Photobacterium* spp. Coral pathogenic *Vibrio* species, e.g., *V. owensii*, *V. rotiferianus* and *V. alginolyticus*, were among the indicated species of the Xisha Islands. *V. owensii*, *V. parahaemolyticus* and *V. rotiferianus* are the potential coral-pathogens that cause diseases of corals [7, 24]. *V. alginolyticus* and *P. rosenbergii* are detected from unhealthy corals at Tioman Island Marine Park, Malaysia [82]. The relative abundances of these coral pathogenic vibrios in the Xisha Islands are higher than those in the Daya Bay (Fig. 3d). The 2020 coral bleaching event in the Xisha Islands may cause the increasements of coral pathogenic vibrios [63]. With high coral number, *Vibrio* in the Xisha Islands may be developed as the dominant group by influencing coral health. In sum, the distribution patterns of *Vibrio* spp. reflected differences between coral poor and rich areas.

### Distinct community structure of* Vibrio* spp. may be affected by biological factors and assembly mechanism

Previous reports on community structure of *Vibrio* mainly focused on the influence of physicochemical parameters, showing that temperature and salinity are important factors [38, 45]. However, the interaction between *Vibrio* and corals may play an important role in coral reef areas. In the Daya Bay, the main *Vibrio* species showed a positive correlation with corals (Fig. 5a), which may be due to the fact that corals provide nutrients for their growth [56]. The community structure of *Vibrio* in the Xisha Islands was greatly affected by corals (Fig. 4c), and the main groups showed negatively correlation with coral (Fig. 5b). The Xisha Islands face the problem of coral bleaching, and there is a close relationship between coral bleaching and *Vibrio*, perhaps causing a negative correlation of them [32, 63]. Meanwhile, vibrios in Xisha Islands were driven by ecological drift, whereas that in Daya Bay was mainly influenced by ecological drift and homogenizing selection (Fig. 6c), which might be related to the habitat filtration. Habitat filtering across the globe has been reported to cause the various structure of marine bacterial communities [83]. Ecological drift, used to describe random fluctuations in species abundance over time, is a process that can lead to different microbial communities between host species as these communities apart from each other over evolutionary time [84]. For instance, ecological drift drives interindividual diversification of gut microbes in early infants, causing an increase in β-diversity of microbial community [85]. Meanwhile, microbes in coral ecosystems are diverse, and even within the same coral individual, the microbial communities are different [86]. High β-diversity of *Vibrio* communities was also discovered in the Xisha Islands (Additional file 1: Fig. S4). Thus, the large number of different corals species in the Xisha Islands may have influenced the surrounding seawater to produce a distinct *Vibrio* community composition, making ecological drift a major community assembly process. Homogenizing selection refers to the selective effect of similar environment on the formation of microbial community, and leads to the convergence of microbes [87]. Lower β-diversity of *Vibrio* in the Daya Bay (Additional file 1: Fig. S4) indicated that *Vibrio* community tended to be more consistent than that in Xisha Islands. Furthermore, it has been found that more similar environmental conditions in smaller spatial ranges can promote the effect of homogenizing selection [88]. Thus, the less number and species of corals and the relatively small spatial range in the Daya Bay may provide a relatively single living environment for *Vibrio* spp., which may weaken the ecological drift and enhance the homogenizing selection.

## Conclusion

We studied the distribution pattern and assembly mechanism of *Vibrio* spp. in both coral rich (Xisha Islands) and poor areas (Daya Bay) by molecular approaches. The abundance of *Vibrio* in the Xisha Islands was significantly higher than that in Daya Bay, which may be related to the interaction with corals. Community composition analysis showed that coral might stimulate the growth of the whole *Vibrio* genus resulting in less species specificity, in which *V. campbellii*, *Paraphotobacterium marinum* and *V. caribbeanicus* with strong environmental adaptability were dominant in both two areas. However, there were still heterogeneity of these two areas as assessed through random forest. The indicator species of coral poor areas were mainly *Photobacterium* spp., whereas those in coral rich areas were dominated by *Vibrio* spp. The assembly mechanisms of *Vibrio* communities appeared dissimilarly between the two regions. In the coral poor areas, homogenizing selection and ecological drift dominated the assembly of *Vibrio* communities, whereas in the coral rich areas, only ecological drift dominated the assembly of *Vibrio* communities. Different number and species of corals, environmental (e.g., temperature) and spatial factors between these two coral reef environments may be the primary causes. However, our study only assessed the dynamics of *Vibrio* in the coral environments by molecular approaches, and the possible colonization status of *Vibrio* in corals should be further evaluated experimentally to enrich the background data of coral reef protection.

### Supplementary Information

**Additional file 1:** Supplementary Material Figs. S1-S6, Tables S1-S5.

## Data Availability

All sequencing data were stored in the National Center for Biotechnology Information (NCBI) Sequence Read Archive (SRA), with the accession numberSRP420698 and bioproject number PRJNA930676.

## References

[1] Hoegh-Guldberg O, Poloczanska ES, Skirving W, Dove S (2017). Coral reef ecosystems under climate change and ocean acidification. Front Mar Sci.

[2] Nelson HR, Kuempel CD, Altieri AH (2016). The resilience of reef invertebrate biodiversity to coral mortality. Ecosphere.

[3] MacNeil MA, Graham NAJ, Cinner JE, Wilson SK, Williams ID, Maina J, Newman S, Friedlander AM, Jupiter S, Polunin NVC, McClanahan TR (2015). Recovery potential of the world's coral reef fishes. Nature.

[4] Stat M, Morris E, Gates RD (2008). Functional diversity in coral–dinoflagellate symbiosis. Proc Natl Acad Sci USA.

[5] Hoppe H-G, Schramm W, Bacolod P (1988). Spatial and temporal distribution of pelagic microorganisms and their proteolytic activity over a partly destroyed coral reef. Mar Ecol Prog Ser.

[6] Kelly LW, Williams GJ, Barott KL, Carlson CA, Dinsdale EA, Edwards RA, Haas AF, Haynes M, Lim YW, McDole T, Nelson CE, Sala E, Sandin SA, Smith JE, Vermeij MJA, Youle M, Rohwer F (2014). Local genomic adaptation of coral reef-associated microbiomes to gradients of natural variability and anthropogenic stressors. Proc Natl Acad Sci USA.

[7] Kemp KM, Westrich JR, Alabady MS, Edwards ML, Lipp EK (2017). Abundance and multilocus sequence analysis of *Vibrio* associated with diseased elkhorn coral, *Acropora palmata*, of the Florida Keys. Appl Environ Microb.

[8] Galand PE, Ruscheweyh H-J, Salazar G, Hochart C, Henry N, Hume BCC (2023). Diversity of the Pacific Ocean coral reef microbiome. Nat Commun.

[9] Shnit-Orland M, Kushmaro A (2009). Coral mucus-associated bacteria: a possible first line of defense. FEMS Microbiol Ecol.

[10] Rädecker N, Pogoreutz C, Voolstra CR, Wiedenmann J, Wild C (2015). Nitrogen cycling in corals: the key to understanding holobiont functioning?. Trends Microbiol.

[11] Sun W, Anbuchezhian R, Li Z, Goffredo S, Dubinsky Z (2016). Association of coral-microbes, and the ecological roles of microbial symbionts in corals. The cnidaria, past, present and future.

[12] Taniguchi A, Yoshida T, Eguchi M (2014). Bacterial production is enhanced by coral mucus in reef systems. J Exp Mar Biol Ecol.

[13] Tanaka Y, Ogawa H, Miyajima T (2011). Bacterial decomposition of coral mucus as evaluated by long-term and quantitative observation. Coral Reefs.

[14] Nakajima R, Yoshida T, Azman BAR, Zaleha K, Othman BHR, Toda T (2009). In situ release of coral mucus by *Acropora* and its influence on the heterotrophic bacteria. Aquat Ecol.

[15] Nelson CE, Goldberg SJ, Wegley Kelly L, Haas AF, Smith JE, Rohwer F, Carlson CA (2013). Coral and macroalgal exudates vary in neutral sugar composition and differentially enrich reef bacterioplankton lineages. ISME J.

[16] Takemura A, Chien D, Polz M (2014). Associations and dynamics of *Vibrionaceae* in the environment, from the genus to the population level. Front Microbiol.

[17] Tout J, Jeffries TC, Webster NS, Stocker R, Ralph PJ, Seymour JR (2014). Variability in microbial community composition and function between different niches within a coral reef. Microb Ecol.

[18] Weber L, Apprill A (2020). Diel, daily, and spatial variation of coral reef seawater microbial communities. PLoS ONE.

[19] Xu W, Gong L, Yang S, Gao Y, Ma X, Xu L, Chen H, Luo Z (2020). Spatiotemporal dynamics of *Vibrio* communities and abundance in Dongshan Bay, South of China. Front Microbiol.

[20] Thompson JR, Randa MA, Marcelino LA, Tomita-Mitchell A, Lim E, Polz MF (2004). Diversity and dynamics of a north Atlantic coastal *Vibrio* community. Appl Environ Microb.

[21] Zhang X-H, Lin H, Wang X, Austin B (2018). Significance of *Vibrio* species in the marine organic carbon cycle. A review. Sci China Earth Sci.

[22] Fu J, Li Y, Zhao L, Wu C, He Z (2023). Characterization and genomic analysis of a bacteriophage with potential in lysing *Vibrio alginolyticus*. Viruses.

[23] Pfeifer E, Michniewski S, Gätgens C, Münch E, Müller F, Polen T, Millard A, Blombach B, Frunzke J (2019). Generation of a prophage-free variant of the fast-growing bacterium *Vibrio natriegens*. Appl Environ Microb.

[24] Amin AKMR, Feng G, Al-saari N, Meirelles PM, Yamazaki Y, Mino S, Thompson FL, Sawabe T, Sawabe T (2016). The first temporal and spatial assessment of *Vibrio* diversity of the surrounding seawater of coral reefs in Ishigaki, Japan. Front Microbiol.

[25] Couce E, Ridgwell A, Hendy EJ (2012). Environmental controls on the global distribution of shallow-water coral reefs. J Biogeogr.

[26] Weber L, Gonzalez-Díaz P, Armenteros M, Apprill A (2019). The coral ecosphere: a unique coral reef habitat that fosters coral–microbial interactions. Limnol Oceanogr.

[27] Fu X, Wang C, Shao C, Han L, Li G, Zeng X, Guan H (2009). Investigation on the status of coral reef resources and medicinal research in China I. Coral reef resources and ecological functions. J Ocean U China.

[28] Yu K (2012). Coral reefs in the South China Sea: their response to and records on past environmental changes. Sci China Earth Sci.

[29] Huang H, You F, Lian J, Yang J, Li X, Dong Z, Zhang C, Yuan T (2011). Species diversity and distribution of scleractinian coral at Xisha Islands, China. Biodivers Sci.

[30] Zhao M, Yu K, Shi Q, Chen T, Zhang H, Chen T (2013). Coral communities of the remote atoll reefs in the Nansha Islands, southern South China Sea. Environ Monit Assess.

[31] Zuo X, Su F, Zhao H, Zhang J, Wang Q, Wu D (2017). Regional hard coral distribution within geomorphic and reef flat ecological zones determined by satellite imagery of the Xisha Islands, South China Sea. Chin J Ocean Limnol.

[32] Xiao J, Wang W, Wang X, Tian P, Niu W (2022). Recent deterioration of coral reefs in the South China Sea due to multiple disturbances. PeerJ.

[33] Zhao J, Qiu Y, Zhang H, Cheng T (2007). Distribution and ecological characters of neritic stone corals at Daya Bay. J Trop Oceanogr.

[34] Li S, Yu K, Shi Q, Chen T, Zhao M, Zhao J (2008). Interspecies and spatial diversity in the symbiotic zooxanthellae density in corals from northern South China Sea and its relationship to coral reef bleaching. Chin Sci Bull.

[35] Guo F, Xiao J, Tian P, Wang W, Wang X, Huang D, Wang J, Niu W (2022). Status of scleractinian corals in Daya Bay and along the coast of Dapeng Peninsula and ecological vulnerability assessment. J Appl Oceanogr.

[36] Shi R, Xu S, Qi Z, Zhu Q, Huang H, Weber F (2019). Influence of suspended mariculture on vertical distribution profiles of bacteria in sediment from Daya Bay, Southern China. Mar Pollut Bull.

[37] Zhang X, Zhang Z, Li J, Lu Y, Zheng P, Xian J (2022). Isolation and identification of marine Vibrio from Xisha Yongle Islands, China. J Fish Res.

[38] Wang X, Liu J, Liang J, Sun H, Zhang X-H (2020). Spatiotemporal dynamics of the total and active *Vibrio* spp. populations throughout the Changjiang estuary in China. Environ Microbiol.

[39] Bohlen L, Dale AW, Sommer S, Mosch T, Hensen C, Noffke A, Scholz F, Wallmann K (2011). Benthic nitrogen cycling traversing the Peruvian oxygen minimum zone. Geochim Cosmochim Acta.

[40] Wang J, Yao P, Bianchi TS, Li D, Zhao B, Cui X, Pan H, Zhang T, Yu Z (2015). The effect of particle density on the sources, distribution, and degradation of sedimentary organic carbon in the Changjiang Estuary and adjacent shelf. Chem Geol.

[41] Zhao B, Yao P, Li D, Yu Z (2021). Effects of river damming and delta erosion on organic carbon burial in the Changjiang Estuary and adjacent East China Sea inner shelf. Sci Total Environ.

[42] Church C, Spargo J, Fishel S (2017). Strong acid extraction methods for "total phosphorus" in soils: EPA method 3050B and EPA method 3051. Agric Environ Lett.

[43] Varol M, Sen B (2012). Assessment of nutrient and heavy metal contamination in surface water and sediments of the upper Tigris River, Turkey. CATENA.

[44] Vezzulli L, Brettar I, Pezzati E, Reid PC, Colwell RR, Höfle MG, Pruzzo C (2012). Long-term effects of ocean warming on the prokaryotic community: evidence from the vibrios. ISME J.

[45] Liang J, Liu J, Wang X, Lin H, Liu J, Zhou S, Sun H, Zhang X-H (2019). Spatiotemporal dynamics of free-living and particle-associated vibrio communities in the Northern Chinese Marginal Seas. Appl Environ Microb.

[46] Chen S, Zhou Y, Chen Y, Gu J (2018). fastp: an ultra-fast all-in-one FASTQ preprocessor. Bioinformatics.

[47] Magoč T, Salzberg SL (2011). FLASH: fast length adjustment of short reads to improve genome assemblies. Bioinformatics.

[48] Callahan BJ, McMurdie PJ, Rosen MJ, Han AW, Johnson AJA, Holmes SP (2016). DADA2: high-resolution sample inference from Illumina amplicon data. Nat Methods.

[49] Caporaso JG, Kuczynski J, Stombaugh J, Bittinger K, Bushman FD, Costello EK (2010). QIIME allows analysis of high-throughput community sequencing data. Nat Methods.

[50] ter Braak CJF, Smilauer P. Canoco reference manual and user's guide: software for ordination, version 5.0. Microcomputer Power. 2012.

[51] Stegen JC, Lin X, Konopka AE, Fredrickson JK (2012). Stochastic and deterministic assembly processes in subsurface microbial communities. ISME J.

[52] Kooperman N, Ben-Dov E, Kramarsky-Winter E, Barak Z, Kushmaro A (2007). Coral mucus-associated bacterial communities from natural and aquarium environments. FEMS Microbiol Lett.

[53] Schöttner S, Hoffmann F, Wild C, Rapp HT, Boetius A, Ramette A (2009). Inter- and intra-habitat bacterial diversity associated with cold-water corals. ISME J.

[54] Li N, Dong K, Jiang G, Tang J, Xu Q, Li X, Kang Z, Zou S, Chen X, Adams JM, Zhao H (2020). Stochastic processes dominate marine free-living Vibrio community assembly in a subtropical gulf. FEMS Microbiol Ecol.

[55] Wang X, Liu J, Zhao W, Liu J, Liang J, Thompson F, Zhang X-H (2022). Fine-scale structuring of planktonic *Vibrio* spp. in the Chinese marginal seas. Appl Environ Microb.

[56] Brown BE, Bythell JC (2005). Perspectives on mucus secretion in reef corals. Mar Ecol Prog Ser.

[57] Garren M, Azam F (2010). New method for counting bacteria associated with coral mucus. Appl Environ Microb.

[58] Allers E, Niesner C, Wild C, Pernthaler J (2008). Microbes enriched in seawater after addition of coral mucus. Appl Environ Microb.

[59] Tait K, Hutchison Z, Thompson FL, Munn CB (2010). Quorum sensing signal production and inhibition by coral-associated vibrios. Environ Microbiol Rep.

[60] Pollock FJ, McMinds R, Smith S, Bourne DG, Willis BL, Medina M, Thurber RV, Zaneveld JR (2018). Coral-associated bacteria demonstrate phylosymbiosis and cophylogeny. Nat Commun.

[61] Niggl W, Glas M, Laforsch C, Mayr C, Wild C. First evidence of coral bleaching stimulating organic matter release by reef corals. In: Proceedings of the 11th international coral reef symposium, Ft. Lauderdale, Florida, 7–11 July 2008, Session 19. 2009.

[62] Radice VZ, Fry B, Dove SG, Hoegh-Guldberg O (2021). Biogeochemical variability and trophic status of reef water column following a coral bleaching event. Coral Reefs.

[63] Zhao W, Chen L, Liu M, Huang K, Ding Y, Xiao J, Tian P, Liu J, Zhang X-H, Niu W, Wang X (2023). Sedimentary Vibrio blooms in the Xisha Islands may associate with the 2020 coral bleaching event. Appl Environ Microb.

[64] Qin Z, Yu K, Liang J, Yao Q, Chen B (2020). Significant changes in microbial communities associated with reef corals in the southern south china sea during the 2015/2016 global-scale coral bleaching event. J Geophys Res Oceans.

[65] Falkowski PG, Dubinsky Z, Muscatine L, Porter JW (1984). Light and the bioenergetics of a symbiotic coral. Bioscience.

[66] Kang SR, Srinivasan S, Lee S-S (2015). *Vibrio oceanisediminis* sp. nov., a nitrogen-fixing bacterium isolated from an artificial oil-spill marine sediment. Int J Syst Evol Microbiol.

[67] Rowan-Nash AD, Korry BJ, Mylonakis E, Belenky P (2019). Cross-domain and viral interactions in the microbiome. Microbiol Mol Biol R.

[68] Shieh WY, Simidu U, Maruyama Y (1990). A photobacterium-like bacterium able to fix nitrogen. Antonie Van Leeuwenhoek.

[69] Yu T, Li M, Niu M, Fan X, Liang W, Wang F (2018). Difference of nitrogen-cycling microbes between shallow bay and deep-sea sediments in the South China Sea. Appl Microbiol Biotechnol.

[70] Liang J, Liu J, Wang X, Sun H, Zhang Y, Ju F, Thompson F, Zhang X-H (2022). Genomic analysis reveals adaptation of *Vibrio campbellii* to the Hadal Ocean. Appl Environ Microb.

[71] Wang L, Chen Y, Huang H, Huang Z, Chen H, Shao Z (2015). Isolation and identification of *Vibrio campbellii* as a bacterial pathogen for luminous vibriosis of *Litopenaeus vannamei*. Aquac Res.

[72] Aunkham A, Suginta W (2021). Probing the physiological roles of the extracellular loops of chitoporin from *Vibrio campbellii*. Biophys J.

[73] Simpson CA, Petersen BD, Haas NW, Geyman LJ, Lee AH, Podicheti R, Pepin R, Brown LC, Rusch DB, Manzella MP, Papenfort K, van Kessel JC (2021). The quorum-sensing systems of *Vibrio campbellii* DS40M4 and BB120 are genetically and functionally distinct. Environ Microbiol.

[74] Barekzi N, Wang Z, Rubin RA, Rahbar AM, Thach DC, Meador CE, Vora GJ. Transcriptomic, proteomic and phenotypic analysis of the *Vibrio campbellii* stationary phase alternative sigma factors RpoS1 and RpoS2.

[75] Huang Z, Dong C, Shao Z (2016). *Paraphotobacterium marinum* gen. nov., sp. nov., a member of the family Vibrionaceae, isolated from surface seawater. Int J Syst Evol Microbiol.

[76] Hoffmann M, Monday SR, Allard MW, Strain EA, Whittaker P, Naum M, McCarthy PJ, Lopez JV, Fischer M, Brown EW (2012). *Vibrio caribbeanicus* sp. nov., isolated from the marine sponge *Scleritoderma cyanea*. Int J Syst Evol Microbiol.

[77] Chen X, Zhao H, Jiang G, Tang J, Xu Q, Huang L, Chen S, Zou S, Dong K, Li N (2020). Responses of free-living vibrio community to seasonal environmental variation in a subtropical Inland Bay. Front Microbiol.

[78] Yoshizawa S, Wada M, Kita-Tsukamoto K, Yokota A, Kogure K (2009). *Photobacterium aquimaris* sp. nov., a luminous marine bacterium isolated from seawater. Int J Syst Evol Microbiol.

[79] Dunlap PV (1985). Osmotic control of luminescence and growth in *Photobacterium leiognathi* from ponyfish light organs. Arch Microbiol.

[80] Liu J, Zhuang H, Liu Y, Pharmacy SO (2014). Detection of muscular fatty acids in 8 ponyfish species in Zhanjiang ocean. J Guangdong Med Coll.

[81] Lin Z, Wang X, Jiang Y (2010). Distribution and species composition of fish eggs in Daya Bay. J Fish Sci China.

[82] Khodzori FA, Saad S, Mansor NN, Nasir NANM, Khalid NNNA, Rawi FZ (2021). Pathogenic *Vibrio* spp. identified for white syndrome coral disease in Tioman Island Marine Park, Malaysia. Malays J Microbiol..

[83] Pontarp M, Canbäck B, Tunlid A, Lundberg P (2012). Phylogenetic analysis suggests that habitat filtering is structuring marine bacterial communities across the globe. Microb Ecol.

[84] Kohl KD (2020). Ecological and evolutionary mechanisms underlying patterns of phylosymbiosis in host-associated microbial communities. Philos Trans R Soc B.

[85] Seki D, Schauberger C, Hausmann B, Berger A, Wisgrill L, Berry D (2022). Individuality of the extremely premature infant gut microbiota is driven by ecological drift. mSystems..

[86] Zhang J, Hu A, Sun Y, Yang Q, Dong J, Long L, Huang S (2021). Dispersal limitation expands the diversity of coral microbiome metacommunity in the South China Sea. Front Mar Sci.

[87] Stegen JC, Lin X, Fredrickson JK, Konopka AE (2015). Estimating and mapping ecological processes influencing microbial community assembly. Front Microbiol.

[88] Logares R, Tesson SVM, Canbäck B, Pontarp M, Hedlund K, Rengefors K (2018). Contrasting prevalence of selection and drift in the community structuring of bacteria and microbial eukaryotes. Environ Microbiol.

