# Supplementary material for: Distinct coral environments shape the dynamic of planktonic Vibrio spp
Source: Environ Microbiome. 2023 Oct 23;18:77. doi: 10.1186/s40793-023-00532-7 (PMC10594878; doi:10.1186/s40793-023-00532-7)
Supplement: Supplementary file 1 — Additional file 1: Supplementary Material Figs. S1-S6, Tables S1-S5. [file 40793_2023_532_MOESM1_ESM.pdf]

Distinct coral environments shape the dynamic of planktonic *Vibrio* spp.

Wenbin Zhao[1,2], Xing Chen[1,2], Ronghua Liu[1,2], Peng Tian[3,4], Wentao Niu[3,4], Xiao-Hua Zhang[1,2], Jiwen Liu[1,2*] and Xiaolei Wang[1,2*]

[1] Frontiers Science Center for Deep Ocean Multispheres and Earth System, and College of Marine Life Sciences, Ocean University of China, Qingdao 266003, China

[2] Institute of Evolution & Marine Biodiversity, Ocean University of China, Qingdao 266100, China

[3] Laboratory of Marine Biodiversity Research, Third Institute of Oceanography, Ministry of Natural Resources, 178 Daxue Road, Xiamen 361005, China

[4] Nansha Islands Coral Reef Ecosystem National Observation and Research Station, Guangzhou 510000, China

[*]Author for correspondence:

Xiaolei Wang

Email: wangxiaolei@ouc.edu.cn

Jiwen Liu

Email: liujiwen@ouc.edu.cn

Running title: Dynamic changes of planktonic *Vibrio* spp.

Supplementary Figures

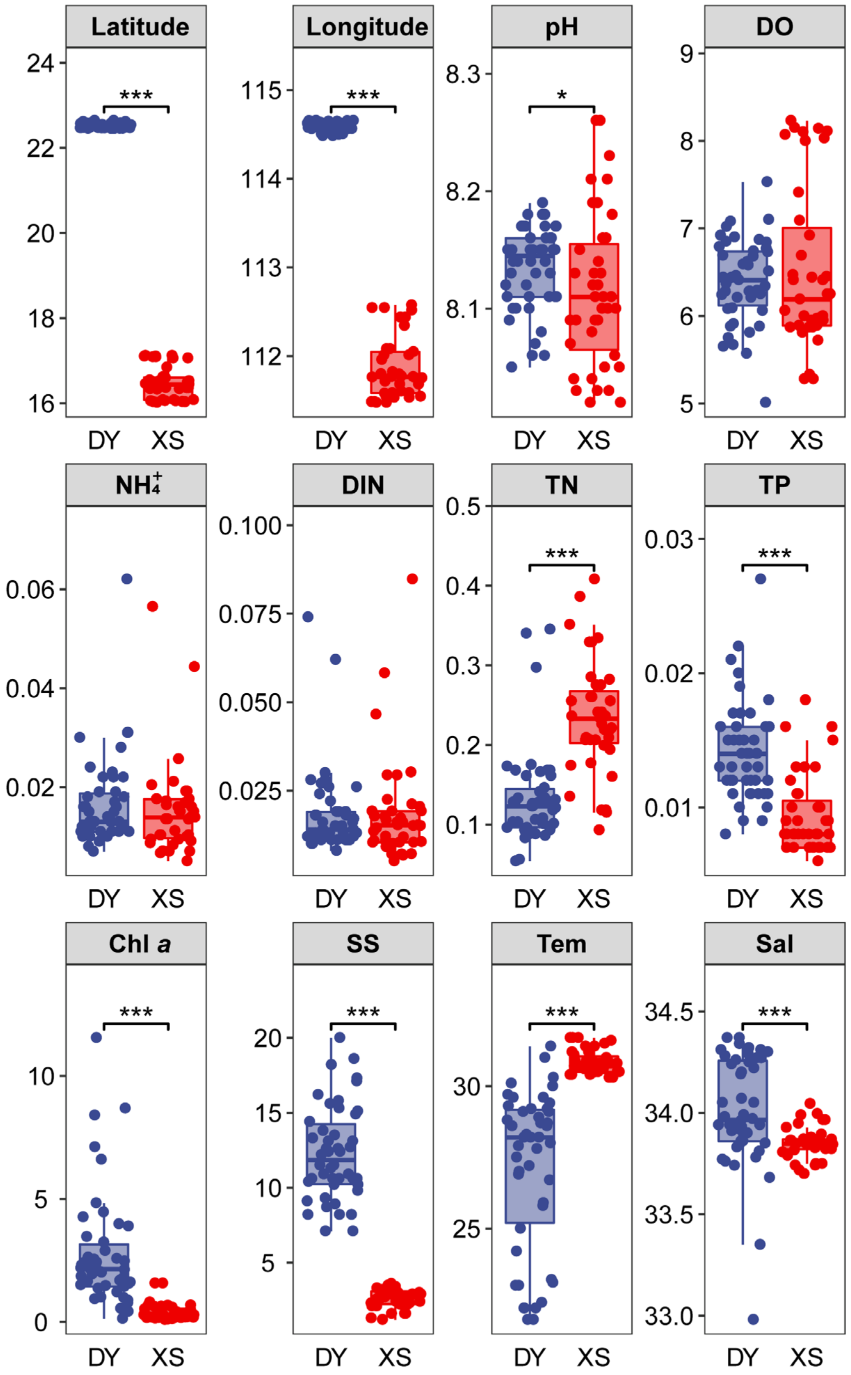

Fig. S1 Environmental parameters in seawater varied significantly between the Daya Bay and Xisha Islands. Dunn's test showed significant differences between Daya Bay and Xisha Islands (*, $P < 0.05$, **, $P < 0.01$, ***, $P < 0.001$). DY, Daya Bay; XS, Xisha Islands. DO, dissolved oxygen; DIN, dissolved inorganic nitrogen; TN, total nitrogen; TP, total phosphorus; Chl *a*, chlorophyll a; SS, suspended solid; Tem, temperature; Sal, salinity.

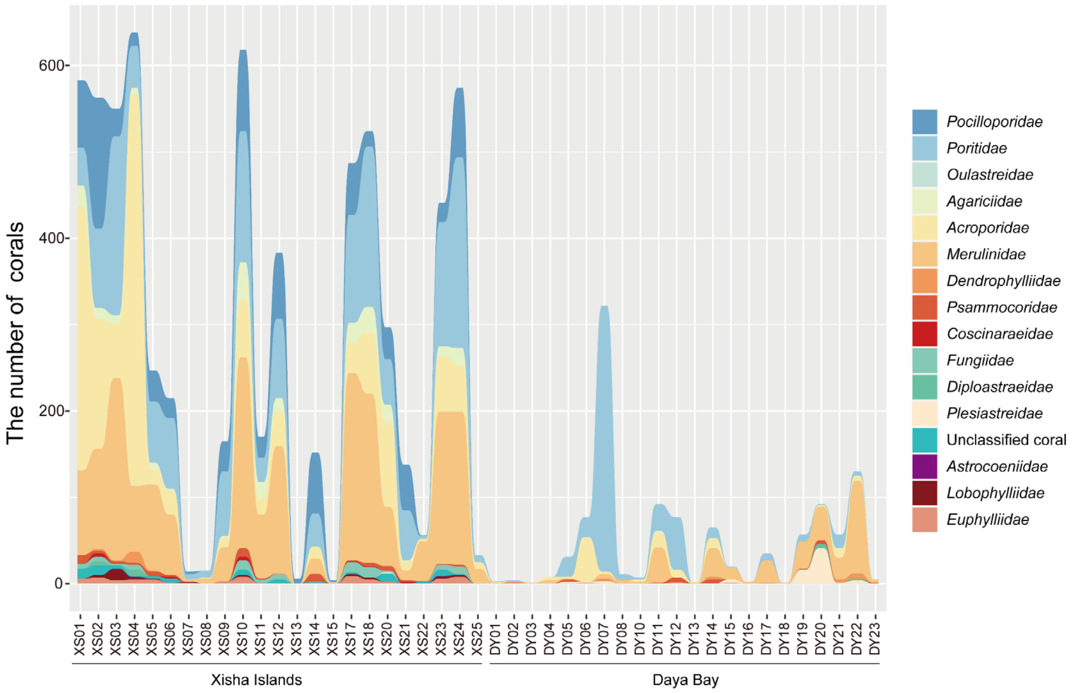

Fig. S2 The distribution of corals between the Daya Bay and Xisha Islands.

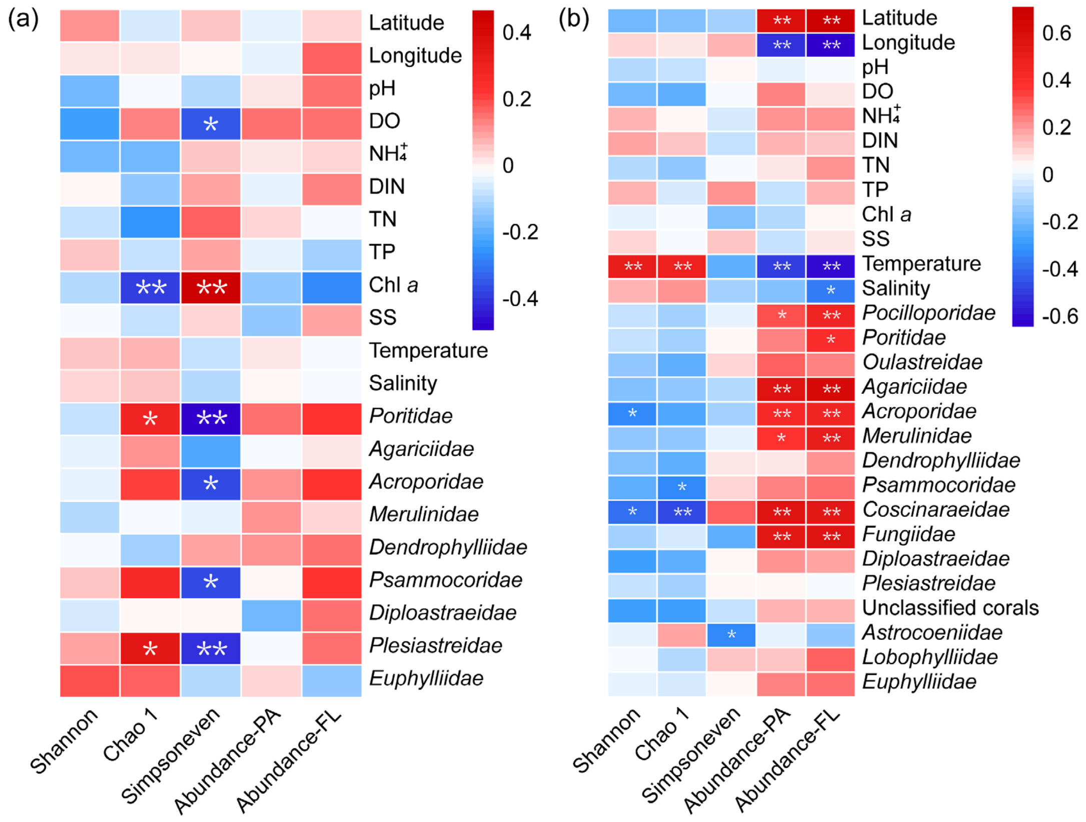

Fig. S3 Spearman correlations between the abundance, α-diversity indices of *Vibrio* spp. and effect factors. (a), Daya Bay; (b), Xisha Islands. *, $P < 0.05$; **, $P < 0.01$.

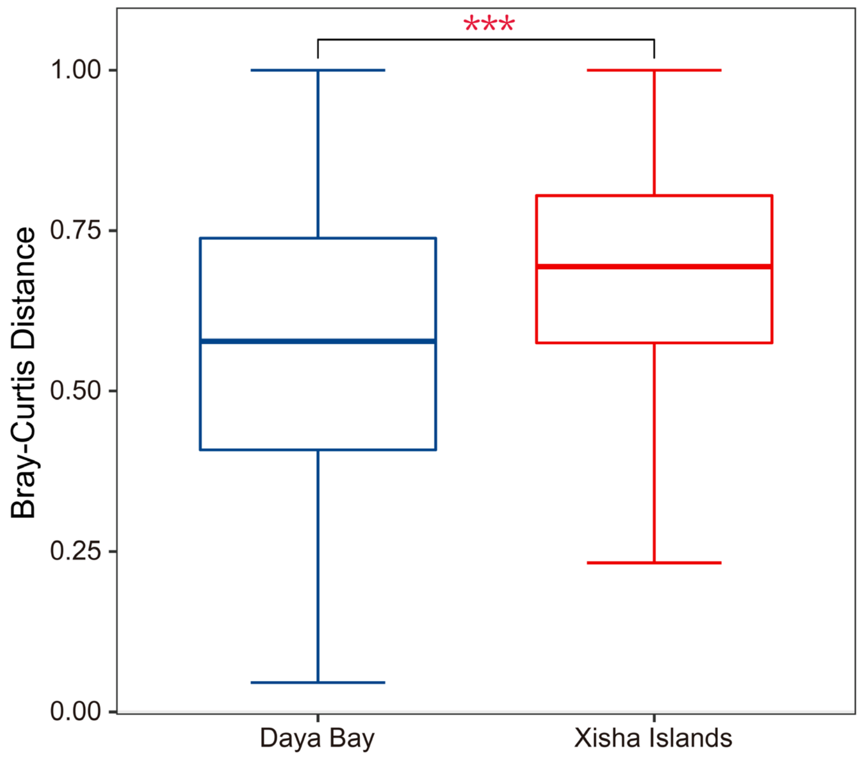

Fig. S4 Bray-Curtis distance represented the β-diversity index. ***, *P* < 0.001

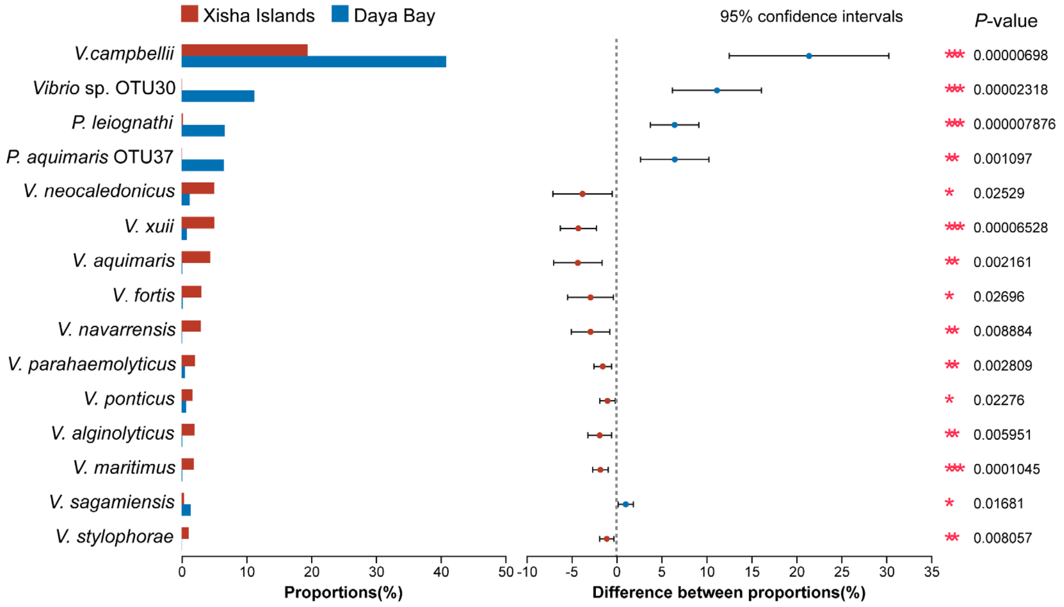

Fig. S5 Different community compositions of *Vibrio* spp. at the species level. *, $P < 0.05$; **, $P < 0.01$; ***, $P < 0.001$.

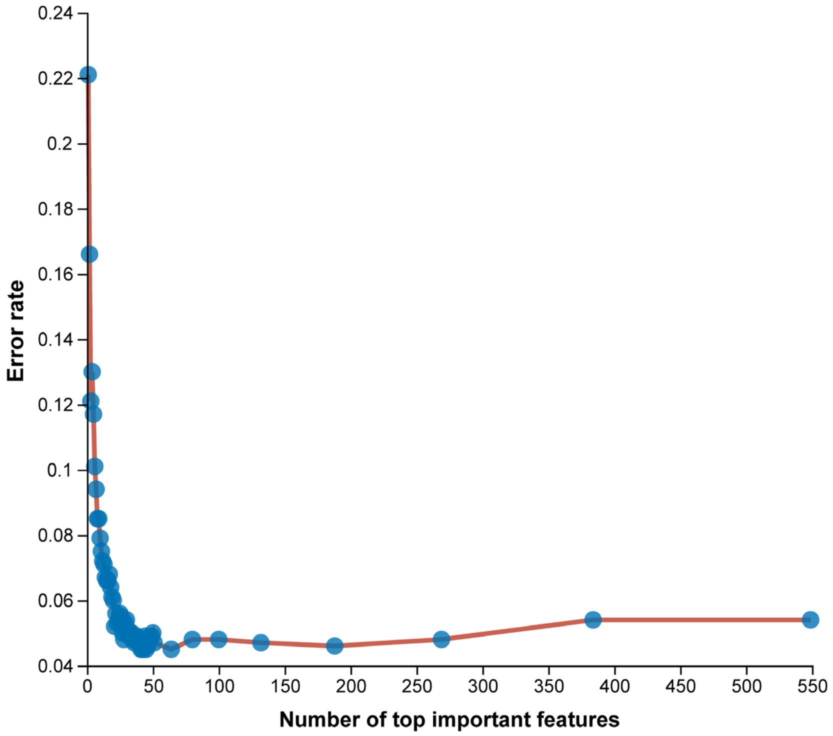

Fig. S6 Predictive accuracy of random forest machine learning. Highest accuracy was achieved with number of top important features = 40 OTUs.

Supplementary Tables

Table S1 Sampling location and the detailed information for samples.

Site	Longitude (°E)	Latitude (°N)	Sampling information	Sampling areas
DY01	114.482	22.479	SW, BW	Daya Bay
DY02	114.495	22.461	SW, BW	Daya Bay
DY03	114.502	22.450	SW, BW	Daya Bay
DY04	114.534	22.466	SW, BW	Daya Bay
DY05	114.560	22.478	SW, BW	Daya Bay
DY06	114.607	22.467	SW, BW	Daya Bay
DY07	114.626	22.452	SW, BW	Daya Bay
DY08	114.640	22.454	SW, BW	Daya Bay
DY09	114.653	22.468	SW, BW	Daya Bay
DY10	114.620	22.471	SW, BW	Daya Bay
DY11	114.592	22.490	SW, BW	Daya Bay
DY12	114.610	22.502	SW, BW	Daya Bay
DY13	114.623	22.518	SW, BW	Daya Bay
DY14	114.605	22.537	SW, BW	Daya Bay
DY15	114.593	22.544	SW, BW	Daya Bay
DY16	114.574	22.548	SW, BW	Daya Bay
DY17	114.562	22.556	SW, BW	Daya Bay
DY18	114.522	22.588	SW, BW	Daya Bay
DY19	114.643	22.638	SW, BW	Daya Bay
DY20	114.632	22.614	SW, BW	Daya Bay
DY21	114.644	22.583	SW, BW	Daya Bay
DY22	114.633	22.572	SW, BW	Daya Bay
DY23	114.650	22.565	SW, BW	Daya Bay
XS01	111.542	17.084	SW, BW	Xisha Islands
XS02	111.482	17.057	SW, BW	Xisha Islands
XS03	111.474	17.101	SW, BW	Xisha Islands
XS04	111.530	17.118	SW, BW	Xisha Islands
XS05	111.760	16.456	SW, BW	Xisha Islands
XS06	112.543	16.076	SW, BW	Xisha Islands
XS07	112.435	16.028	SW, BW	Xisha Islands
XS08	112.515	16.014	SW	Xisha Islands

XS09	112.574	16.051	SW	Xisha Islands
XS10	111.578	16.488	SW	Xisha Islands
XS11	111.601	16.475	SW	Xisha Islands
XS12	111.583	16.441	SW, BW	Xisha Islands
XS13	111.797	16.074	SW, BW	Xisha Islands
XS14	111.820	16.049	SW	Xisha Islands
XS15	111.772	16.032	SW	Xisha Islands
XS16	111.751	16.054	SW	Xisha Islands
XS17	111.680	16.594	SW, BW	Xisha Islands
XS18	111.764	16.543	SW, BW	Xisha Islands
XS19	111.795	16.523	SW	Xisha Islands
XS20	112.342	16.842	SW	Xisha Islands
XS21	111.713	16.616	SW	Xisha Islands
XS22	111.957	16.323	SW	Xisha Islands
XS23	112.010	16.362	SW, BW	Xisha Islands
XS24	112.079	16.356	SW, BW	Xisha Islands
XS25	112.046	16.334	SW, BW	Xisha Islands

SW, surface seawater; BW, bottom seawater.

Table S2 Environmental parameters among the whole sampling sites.

Sample	pH	DO (mg L^{-1})	NH_4^+ (μmol L^{-1})	DIN (μmol L^{-1})	TN (mg L^{-1})	TP (mg L^{-1})	Chl *a* (μg L^{-1})	SS (mg L^{-1})	Tem (℃)	Sal (PSU)
DY01B	8.160	6.290	0.024	0.024	0.175	0.011	0.130	7.100	25.800	34.240
DY01S	8.110	6.280	0.015	0.015	0.123	0.008	1.500	8.200	27.900	33.760
DY02B	8.170	5.880	0.013	0.014	0.123	0.012	2.430	8.200	22.200	34.270
DY02S	8.140	6.410	0.013	0.013	0.088	0.010	1.560	7.100	28.200	33.860
DY03B	8.160	5.880	0.022	0.028	0.088	0.011	4.830	9.800	22.200	34.280
DY03S	8.110	6.280	0.009	0.011	0.091	0.014	4.460	8.900	28.200	33.350
DY04B	8.160	5.650	0.011	0.018	0.083	0.020	8.400	10.600	22.200	34.310
DY04S	8.150	6.740	0.014	0.014	0.086	0.012	2.620	12.200	25.900	34.340
DY05B	8.130	6.580	0.011	0.011	0.096	0.011	1.470	12.700	27.500	34.370
DY05S	8.120	6.590	0.013	0.013	0.091	0.014	1.430	13.300	28.300	34.050
DY06B	8.180	7.020	0.013	0.013	0.128	0.009	1.640	18.600	25.000	34.250
DY06S	8.150	6.920	0.022	0.022	0.103	0.011	1.850	18.200	28.000	33.990
DY07B	8.170	6.900	0.019	0.019	0.093	0.012	3.460	17.100	23.100	34.370
DY07S	8.160	6.870	0.017	0.017	0.128	0.011	3.980	14.900	27.000	33.940
DY08B	8.190	6.790	0.011	0.011	0.101	0.012	3.240	12.600	23.000	34.290
DY08S	8.160	6.770	0.008	0.008	0.106	0.013	2.470	10.600	24.200	34.270
DY09B	8.150	5.750	0.007	0.026	0.093	0.016	7.110	17.300	21.800	34.310
DY09S	8.140	6.680	0.016	0.016	0.054	0.014	0.870	11.500	27.200	34.220

DY10B	8.170	7.080	0.016	0.018	0.146	0.014	2.420	13.100	26.900	34.050
DY10S	8.170	7.100	0.011	0.011	0.108	0.012	1.610	10.400	28.200	34.200
DY11B	8.180	6.620	0.019	0.019	0.161	0.018	2.890	12.400	23.000	34.300
DY11S	8.130	6.440	0.011	0.011	0.118	0.013	1.210	8.700	28.900	33.930
DY12B	8.150	6.850	0.030	0.030	0.056	0.012	1.000	16.200	28.600	33.970
DY12S	8.150	7.530	0.012	0.012	0.121	0.019	0.980	13.100	29.200	33.890
DY13B	8.150	5.010	0.031	0.074	0.166	0.027	8.680	20.000	21.800	34.320
DY13S	8.140	6.440	0.031	0.019	0.133	0.017	4.260	13.800	27.800	33.780
DY14B	8.180	6.340	0.012	0.012	0.103	0.010	2.120	15.100	23.200	34.190
DY14S	8.120	6.510	0.011	0.011	0.168	0.014	2.230	11.500	29.100	33.810
DY15B	8.100	6.090	0.010	0.011	0.131	0.017	3.890	10.200	29.300	33.900
DY15S	8.090	6.060	0.028	0.028	0.126	0.015	2.540	14.400	29.600	33.840
DY16B	8.060	5.670	0.013	0.013	0.345	0.011	2.310	10.600	29.700	33.770
DY16S	8.050	5.570	0.010	0.010	0.168	0.009	1.940	10.200	29.600	33.930
DY17B	8.110	6.210	0.016	0.016	0.340	0.015	1.360	10.600	28.700	33.980
DY17S	8.080	6.240	0.011	0.011	0.098	0.015	2.190	10.600	29.400	33.940
DY18B	8.060	5.910	0.022	0.026	0.297	0.016	1.770	15.800	31.000	33.920
DY18S	8.070	5.810	0.023	0.027	0.173	0.017	2.170	10.900	31.400	33.830
DY19B	8.110	6.210	0.018	0.018	0.133	0.016	0.540	11.300	29.200	33.860
DY19S	8.110	6.080	0.018	0.018	0.141	0.022	0.930	12.400	30.100	33.910
DY20B	8.150	6.410	0.010	0.010	0.096	0.016	0.540	12.600	26.700	34.070

DY20S	8.100	6.280	0.013	0.013	0.101	0.015	0.770	10.900	30.300	33.940
DY21B	8.140	6.370	0.011	0.011	0.103	0.014	0.420	8.200	29.100	33.960
DY21S	8.130	6.250	0.012	0.012	0.128	0.015	1.680	9.300	30.000	33.850
DY22B	8.170	6.690	0.012	0.012	0.161	0.013	2.010	15.600	28.600	33.740
DY22S	8.170	6.730	0.015	0.015	0.128	0.012	2.570	13.500	28.800	33.680
DY23B	8.150	6.460	0.013	0.013	0.168	0.021	6.600	15.300	22.400	34.260
DY23S	8.100	6.840	0.062	0.062	0.128	0.013	11.550	9.100	28.700	32.980
XS01B	8.210	8.140	0.007	0.007	0.221	0.007	0.230	3.400	30.800	33.870
XS01S	8.180	6.690	0.011	0.012	0.241	0.008	0.210	2.100	30.900	33.869
XS02B	8.210	8.150	0.018	0.018	0.282	0.008	0.200	2.900	30.500	33.849
XS02S	8.190	7.090	0.017	0.020	0.238	0.009	0.100	3.200	30.500	33.816
XS03B	8.230	8.230	0.056	0.058	0.216	0.013	0.670	2.900	30.400	33.836
XS03S	8.190	5.810	0.014	0.014	0.194	0.016	0.520	3.500	30.500	33.717
XS04B	8.260	5.890	0.007	0.007	0.206	0.007	0.160	2.400	30.300	33.864
XS04S	8.260	5.890	0.007	0.007	0.206	0.007	0.160	2.400	30.300	33.864
XS05B	8.080	8.000	0.016	0.018	0.386	0.015	0.270	2.400	30.600	33.844
XS05S	8.020	5.330	0.044	0.047	0.351	0.008	0.630	3.000	30.800	33.855
XS06B	8.120	8.100	0.009	0.009	0.219	0.008	0.590	2.400	30.500	33.995
XS06S	8.100	5.950	0.005	0.005	0.177	0.007	0.390	1.600	30.700	33.862
XS07B	8.150	8.110	0.010	0.010	0.160	0.007	0.180	1.600	30.800	33.868
XS07S	8.090	5.870	0.010	0.010	0.226	0.008	0.140	3.100	30.800	33.864

XS08S	8.160	5.970	0.013	0.013	0.334	0.007	0.280	1.300	31.400	33.879
XS09S	8.160	5.980	0.007	0.007	0.209	0.007	0.620	2.100	30.700	33.894
XS10S	8.020	6.060	0.015	0.015	0.285	0.009	0.290	2.200	30.300	33.789
XS11S	8.040	6.450	0.017	0.017	0.233	0.011	0.190	2.200	30.600	33.848
XS12B	8.030	6.250	0.018	0.029	0.236	0.010	0.680	2.300	30.900	33.821
XS12S	8.030	6.250	0.018	0.029	0.236	0.010	0.680	2.300	30.900	33.821
XS13B	8.130	8.070	0.020	0.085	0.206	0.007	0.560	3.300	30.900	33.965
XS13S	8.040	5.870	0.010	0.010	0.255	0.008	0.800	1.600	31.600	34.045
XS14S	8.050	5.720	0.009	0.012	0.115	0.016	0.180	2.900	31.100	33.965
XS15S	8.120	6.920	0.015	0.022	0.093	0.010	0.140	2.600	31.400	33.947
XS16S	8.060	6.470	0.014	0.017	0.174	0.006	0.090	2.100	31.700	33.989
XS17B	8.130	5.890	0.016	0.016	0.260	0.008	0.200	3.000	30.400	33.847
XS17S	8.130	5.890	0.016	0.016	0.260	0.008	0.200	3.000	30.400	33.847
XS18B	8.110	8.030	0.015	0.015	0.199	0.008	0.510	3.200	30.600	33.826
XS18S	8.090	6.190	0.021	0.021	0.255	0.007	0.120	2.300	30.700	33.806
XS19S	8.140	7.410	0.026	0.030	0.241	0.011	0.140	2.900	31.500	33.848
XS20S	8.030	5.870	0.008	0.011	0.408	0.012	0.220	1.200	30.700	33.927
XS21S	8.070	6.440	0.010	0.010	0.209	0.008	0.270	3.100	30.500	33.847
XS22S	8.050	5.950	0.019	0.019	0.135	0.018	0.280	3.600	31.000	33.699
XS23B	8.090	6.000	0.019	0.019	0.118	0.009	1.570	3.100	31.700	33.848
XS23S	8.090	6.000	0.019	0.019	0.118	0.009	1.570	3.100	31.700	33.848

XS24B	8.100	5.280	0.009	0.011	0.275	0.013	0.160	2.700	31.200	33.741
XS24S	8.100	5.280	0.009	0.011	0.275	0.013	0.160	2.700	31.200	33.741
XS25B	8.110	6.410	0.014	0.015	0.329	0.007	0.180	3.100	30.700	33.749
XS25S	8.110	6.410	0.014	0.015	0.329	0.007	0.180	3.100	30.700	33.749

DO, dissolved oxygen; DIN, dissolved inorganic nitrogen; TN, total nitrogen; TP, total phosphorus; Chl *a*, chlorophyll *a*; SS, suspended solid; Tem, temperature; Sal, salinity.

Table S3 Number of corals at the family level among all the sites.

Sample	*Pocilloporidae*	*Poritidae*	*Oulastreidae*	*Agariciidae*	*Acroporidae*	*Merulinidae*	*Dendrophylliidae*	*Psammocoridae*	*Coscinaraeidae*	*Fungiidae*	*Diploastraeidae*	*Plesiastreidae*	Unclassified corals	*Astrocoeniidae*	*Lobophylliidae*	*Euphylliidae*
DY01B	0	1	0	0	1	1	0	0	0	0	0	0	0	0	0	0
DY01S	0	1	0	0	1	1	0	0	0	0	0	0	0	0	0	0
DY02B	0	1	0	0	0	1	0	0	0	0	0	0	0	0	0	2
DY02S	0	1	0	0	0	1	0	0	0	0	0	0	0	0	0	2
DY03B	0	0	0	0	0	1	0	0	0	0	0	0	0	0	0	0
DY03S	0	0	0	0	0	1	0	0	0	0	0	0	0	0	0	0
DY04B	0	0	0	0	4	4	0	0	0	0	0	0	0	0	0	0
DY04S	0	0	0	0	4	4	0	0	0	0	0	0	0	0	0	0
DY05B	0	23	0	0	2	1	0	3	0	0	0	2	0	0	0	0
DY05S	0	23	0	0	2	1	0	3	0	0	0	2	0	0	0	0
DY06B	0	23	0	0	52	0	0	0	0	0	0	1	0	0	0	1
DY06S	0	23	0	0	52	0	0	0	0	0	0	1	0	0	0	1
DY07B	0	308	0	0	3	5	3	0	0	0	0	3	0	0	0	0
DY07S	0	308	0	0	3	5	3	0	0	0	0	3	0	0	0	0
DY08B	0	7	0	0	0	4	0	0	0	0	0	0	0	0	0	0
DY08S	0	7	0	0	0	4	0	0	0	0	0	0	0	0	0	0
DY09B	0	0	0	0	0	0	0	0	0	0	0	0	0	0	0	0
DY09S	0	0	0	0	0	0	0	0	0	0	0	0	0	0	0	0
DY10B	0	2	0	0	3	2	0	0	0	0	0	0	0	0	0	0
DY10S	0	2	0	0	3	2	0	0	0	0	0	0	0	0	0	0
DY11B	0	31	0	2	17	41	0	1	0	0	0	0	0	0	0	0
DY11S	0	31	0	2	17	41	0	1	0	0	0	0	0	0	0	0
DY12B	0	61	0	0	9	0	0	6	0	0	0	1	0	0	0	0
DY12S	0	61	0	0	9	0	0	6	0	0	0	1	0	0	0	0
DY13B	0	0	0	0	1	1	0	0	0	0	0	0	0	0	0	0
DY13S	0	0	0	0	1	1	0	0	0	0	0	0	0	0	0	0
DY14B	0	12	0	0	12	33	3	5	0	0	0	0	0	0	0	0
DY14S	0	12	0	0	12	33	3	5	0	0	0	0	0	0	0	0
DY15B	0	1	0	0	0	14	0	0	0	0	0	4	0	0	0	1
DY15S	0	1	0	0	0	14	0	0	0	0	0	4	0	0	0	1

DY16B	0	0	0	0	0	2	0	0	0	0	0	0	0	0	0	0
DY16S	0	0	0	0	0	2	0	0	0	0	0	0	0	0	0	0
DY17B	0	8	0	0	0	26	1	0	0	0	0	0	0	0	0	0
DY17S	0	8	0	0	0	26	1	0	0	0	0	0	0	0	0	0
DY18B	0	0	0	0	0	1	0	0	0	0	0	0	0	0	0	0
DY18S	0	0	0	0	0	1	0	0	0	0	0	0	0	0	0	0
DY19B	0	8	0	0	0	32	0	1	0	0	0	16	0	0	0	0
DY19S	0	8	0	0	0	32	0	1	0	0	0	16	0	0	0	0
DY20B	0	1	0	0	2	39	0	4	0	0	5	41	0	0	0	0
DY20S	0	1	0	0	2	39	0	4	0	0	5	41	0	0	0	0
DY21B	0	16	0	1	10	25	4	0	0	0	0	1	0	0	0	0
DY21S	0	16	0	1	10	25	4	0	0	0	0	1	0	0	0	0
DY22B	0	5	0	0	6	107	6	0	0	0	2	4	0	0	0	0
DY22S	0	5	0	0	6	107	6	0	0	0	2	4	0	0	0	0
DY23B	0	0	0	0	0	4	0	1	0	0	0	0	0	0	0	0
DY23S	0	0	0	0	0	4	0	1	0	0	0	0	0	0	0	0
XS01B	78	44	0	24	306	98	0	10	0	6	0	0	11	0	0	6
XS01S	78	44	0	24	306	98	0	10	0	6	0	0	11	0	0	6
XS02B	152	92	0	13	150	116	2	3	4	5	5	0	11	0	3	7
XS02S	152	92	0	13	150	116	2	3	4	5	5	0	11	0	3	7
XS03B	32	207	0	11	62	211	1	3	0	2	0	0	4	0	13	4
XS03S	32	207	0	11	62	211	1	3	0	2	0	0	4	0	13	4
XS04B	15	49	0	8	453	76	13	2	0	6	7	0	1	1	2	5
XS04S	15	49	0	8	453	76	13	2	0	6	7	0	1	1	2	5
XS05B	36	71	0	8	17	100	1	5	0	0	0	0	3	0	1	5
XS05S	36	71	0	8	17	100	1	5	0	0	0	0	3	0	1	5
XS06B	23	82	0	3	27	70	0	2	2	0	0	0	5	0	0	1
XS06S	23	82	0	3	27	70	0	2	2	0	0	0	5	0	0	1
XS07B	3	6	0	0	0	3	0	0	0	0	0	0	0	0	1	1
XS07S	3	6	0	0	0	3	0	0	0	0	0	0	0	0	1	1
XS08S	0	8	0	0	1	6	0	0	0	0	0	0	0	0	0	0
XS09S	35	75	0	3	10	39	0	0	0	0	0	0	0	0	0	3
XS10S	94	152	0	43	67	221	0	10	4	11	0	0	6	0	2	8

XS11S	24	28	0	20	18	74	0	0	1	4	0	0	0	0	0	1
XS12B	76	92	0	16	40	147	1	0	0	6	0	0	5	0	0	0
XS12S	76	92	0	16	40	147	1	0	0	6	0	0	5	0	0	0
XS13B	6	0	0	0	0	0	0	0	0	0	0	0	0	0	0	0
XS13S	6	0	0	0	0	0	0	0	0	0	0	0	0	0	0	0
XS14S	71	38	0	1	13	17	1	8	0	0	0	0	2	0	0	1
XS15S	2	1	0	0	0	1	0	0	0	0	0	0	0	0	0	0
XS16S	0	0	0	0	0	0	0	0	0	0	0	0	0	0	0	0
XS17B	60	125	0	23	35	217	0	1	1	12	0	0	2	0	2	9
XS17S	60	125	0	23	35	217	0	1	1	12	0	0	2	0	2	9
XS18B	18	185	1	29	71	196	0	4	1	12	0	0	0	0	2	5
XS18S	18	185	1	29	71	196	0	4	1	12	0	0	0	0	2	5
XS19S	0	0	0	0	0	0	0	0	0	0	0	0	0	0	0	0
XS20S	37	53	0	20	98	68	1	5	0	2	0	1	10	0	0	2
XS21S	53	58	0	2	10	11	0	2	2	0	0	0	0	0	0	0
XS22S	1	3	0	0	3	46	0	1	0	0	0	0	2	0	0	0
XS23B	22	144	0	12	64	176	0	1	0	6	0	0	7	1	2	6
XS23S	22	144	0	12	64	176	0	1	0	6	0	0	7	1	2	6
XS24B	80	221	0	20	54	177	0	2	0	9	0	0	1	0	2	8
XS24S	80	221	0	20	54	177	0	2	0	9	0	0	1	0	2	8
XS25B	0	8	0	0	8	17	0	0	0	0	0	0	0	0	0	0
XS25S	0	8	0	0	8	17	0	0	0	0	0	0	0	0	0	0

Table S4 The absolute abundance and α-diversity indices of *Vibrio* spp. from all samples.

Sample	FL copies/mL	PA copies/mL	Shannon	Chao 1	Simpsoneven
DY01B	1.16×10^{3}	1.11×10^{3}	0.45	18.50	0.07
DY01S	2.68×10^{3}	2.03×10^{3}	1.69	74.33	0.05
DY02B	3.16×10^{2}	5.03×10^{2}	1.91	31.00	0.21
DY02S	5.74×10^{2}	5.95×10^{2}	1.39	63.50	0.04
DY03B	8.26×10^{2}	6.62×10^{1}	1.36	51.00	0.10
DY03S	5.36×10^{2}	2.82×10^{2}	0.68	8.50	0.27
DY04B	6.43×10^{2}	6.20×10^{2}	2.18	40.00	0.17
DY04S	2.19×10^{2}	2.11×10^{3}	1.39	61.75	0.06
DY05B	6.50×10^{2}	2.41×10^{2}	2.27	142.00	0.03
DY05S	2.84×10^{2}	4.99×10^{2}	0.98	19.33	0.11
DY06B	2.15×10^{3}	2.17×10^{3}	1.65	61.43	0.06
DY06S	4.73×10^{2}	6.02×10^{1}	1.44	31.33	0.11
DY07B	6.85×10^{2}	6.55×10^{2}	1.04	24.14	0.08
DY07S	1.41×10^{3}	1.25×10^{3}	1.55	72.20	0.04
DY08B	5.41×10^{2}	4.86×10^{1}	1.11	30.00	0.18
DY08S	3.09×10^{2}	5.99×10^{2}	1.43	31.20	0.15
DY09B	2.30×10^{3}	5.75×10^{2}	1.97	55.20	0.09
DY09S	2.78×10^{3}	1.24×10^{3}	1.65	44.00	0.10
DY10B	1.65×10^{3}	5.48×10^{2}	1.55	84.11	0.03
DY10S	5.55×10^{2}	9.06×10^{2}	0.42	14.00	0.08
DY11B	1.97×10^{3}	2.08×10^{3}	0.41	17.00	0.07
DY11S	5.40×10^{2}	6.61×10^{1}	1.22	114.00	0.02
DY12B	1.04×10^{3}	5.63×10^{2}	0.93	48.50	0.04
DY12S	1.96×10^{3}	5.93×10^{2}	1.51	234.50	0.01
DY13B	2.88×10^{2}	3.13×10^{1}	0.97	8.00	0.34
DY13S	2.90×10^{2}	6.55×10^{2}	0.91	14.00	0.28
DY14B	1.23×10^{3}	6.47×10^{2}	1.90	32.00	0.17
DY14S	2.04×10^{3}	2.12×10^{3}	1.60	44.00	0.08
DY15B	3.22×10^{2}	1.63×10^{3}	1.74	102.67	0.04
DY15S	6.86×10^{2}	5.69×10^{2}	1.53	42.20	0.10
DY16B	1.26×10^{3}	1.11×10^{3}	1.08	17.00	0.25
DY16S	3.85×10^{2}	3.15×10^{1}	1.35	23.00	0.17

DY17B	6.34×10^{2}	6.80×10^{2}	1.49	33.00	0.11
DY17S	5.71×10^{2}	1.15×10^{3}	0.69	4.00	0.50
DY18B	3.57×10^{2}	2.54×10^{2}	1.99	37.25	0.12
DY18S	5.78×10^{2}	5.13×10^{2}	1.79	16.00	0.36
DY19B	6.16×10^{2}	6.28×10^{2}	1.43	50.00	0.06
DY19S	1.29×10^{3}	1.23×10^{3}	1.54	69.00	0.05
DY20B	9.05×10^{2}	5.26×10^{2}	1.78	78.50	0.06
DY20S	1.43×10^{3}	5.88×10^{2}	1.52	58.33	0.06
DY21B	2.01×10^{3}	1.35×10^{3}	1.77	59.00	0.08
DY21S	2.40×10^{2}	1.25×10^{2}	1.68	47.33	0.08
DY22B	5.83×10^{2}	5.38×10^{2}	1.26	29.00	0.14
DY22S	1.09×10^{3}	6.46×10^{1}	0.41	7.00	0.17
DY23B	4.51×10^{2}	1.07×10^{2}	0.87	12.50	0.14
DY23S	5.82×10^{2}	5.78×10^{2}	1.76	25.00	0.18
XS01B	1.75×10^{3}	1.25×10^{3}	2.02	55.00	0.08
XS01S	2.31×10^{3}	2.66×10^{3}	1.56	35.20	0.07
XS02B	3.93×10^{3}	2.40×10^{3}	2.27	33.33	0.27
XS02S	2.40×10^{3}	2.44×10^{3}	0.70	5.00	0.40
XS03B	5.14×10^{3}	4.42×10^{3}	2.27	52.20	0.14
XS03S	3.91×10^{3}	1.69×10^{1}	2.31	36.00	0.25
XS04B	2.42×10^{3}	2.39×10^{3}	1.12	56.50	0.04
XS04S	2.72×10^{3}	6.84×10^{3}	1.55	25.50	0.14
XS05B	1.78×10^{2}	5.24×10^{1}	1.61	23.00	0.17
XS05S	6.49×10^{2}	1.86×10^{2}	3.22	94.88	0.15
XS06B	4.82×10^{2}	6.20×10^{2}	0.68	5.00	0.39
XS06S	1.10×10^{3}	1.12×10^{3}	0.77	20.00	0.13
XS07B	1.63×10^{1}	6.34×10^{1}	1.98	37.00	0.18
XS07S	5.40×10^{2}	5.09×10^{1}	1.80	31.00	0.18
XS08S	1.38×10^{1}	1.12×10^{1}	1.29	17.33	0.20
XS09S	5.67×10^{2}	5.12×10^{2}	2.80	102.33	0.22
XS10S	7.26×10^{3}	7.66×10^{3}	0.54	13.50	0.11
XS11S	7.95×10^{3}	7.99×10^{3}	2.13	47.00	0.28
XS12B	4.90×10^{3}	3.51×10^{3}	1.02	35.00	0.05
XS12S	4.30×10^{3}	3.08×10^{3}	1.96	40.50	0.11
XS13B	8.93×10^{2}	6.50×10^{2}	2.25	56.33	0.11

XS13S	6.98×10^{2}	1.14×10^{2}	3.31	172.00	0.07
XS14S	9.50×10^{1}	1.42×10^{2}	2.77	52.00	0.25
XS15S	1.58×10^{2}	1.28×10^{2}	2.84	107.20	0.08
XS16S	7.20×10^{1}	1.08×10^{3}	3.29	122.13	0.14
XS17B	7.63×10^{3}	7.11×10^{3}	2.57	69.50	0.13
XS17S	7.17×10^{3}	7.45×10^{3}	1.83	44.25	0.15
XS18B	4.22×10^{3}	5.06×10^{3}	1.60	16.00	0.28
XS18S	6.99×10^{3}	7.29×10^{3}	1.62	34.00	0.12
XS19S	3.41×10^{3}	3.98×10^{3}	2.96	83.67	0.14
XS20S	2.00×10^{3}	1.37×10^{3}	1.76	33.50	0.15
XS21S	8.22×10^{3}	7.20×10^{3}	2.12	46.25	0.12
XS22S	6.33×10^{2}	6.95×10^{2}	1.07	13.00	0.40
XS23B	1.13×10^{2}	1.31×10^{2}	2.91	152.13	0.07
XS23S	1.16×10^{2}	1.35×10^{2}	2.73	90.20	0.11
XS24B	5.93×10^{2}	6.12×10^{2}	3.48	152.20	0.11
XS24S	6.78×10^{2}	6.13×10^{1}	2.92	78.60	0.23
XS25B	1.97×10^{2}	4.39×10^{2}	2.14	57.50	0.11
XS25S	1.45×10^{2}	1.16×10^{2}	1.59	35.00	0.16

PA, particle-associated; FL, free-living.

Table S5 Mantel test for the biotic and abiotic factors.

Characteristic	Daya Bay		Xisha Islands	
	r	*P*-value	r	*P*-value
Latitude	0.180	0.005	0.100	0.055
Longitude	0.420	0.001	0.184	0.013
pH	0.050	0.213	0.073	0.152
DO	0.020	0.357	0.024	0.325
NH_4^+	0.030	0.307	-0.051	0.648
DIN	-0.020	0.491	-0.142	0.946
TN	-0.010	0.499	-0.023	0.584
TP	0.070	0.155	0.033	0.332
Chl *a*	-0.010	0.492	-0.120	0.922
SS	0.050	0.24	0.131	0.054
Temperature	0.070	0.114	0.013	0.381
Salinity	0.070	0.173	-0.013	0.572
Pocilloporidae	-	-	0.238	0.009
Poritidae	-0.080	0.79	0.010	0.408
Oulastreidae	-	-	0.195	0.051
Agariciidae	-0.100	0.885	0.087	0.102
Acroporidae	-0.140	0.978	0.041	0.289
Merulinidae	-0.030	0.577	-0.038	0.75
Dendrophylliidae	-0.130	0.962	0.024	0.341
Psammocoridae	-0.140	0.987	0.024	0.361
Coscinaraeidae	-	-	0.372	0.001
Fungiidae	-	-	0.084	0.094
Diploastraeidae	-0.160	0.993	0.176	0.043
Plesiastreidae	-0.140	0.987	0.090	0.238
Unclassified corals	-	-	0.139	0.056
Astrocoeniidae	-	-	-0.105	0.856
Lobophylliidae	-	-	0.022	0.375
Euphylliidae	0.090	0.15	-0.013	0.565

DO, dissolved oxygen; DIN, dissolved inorganic nitrogen; TN, total nitrogen; TP, total phosphorus; Chl *a*, chlorophyll *a*; SS, suspended solid. -, no data.
